# Fe_3_O_4_/Pd NPs immobilized on triazine-based polyurethane microspheres as a magnetically recoverable catalyst for the reduction of nitroarenes

**DOI:** 10.1039/d6na00220j

**Published:** 2026-06-19

**Authors:** Sindhu I. Sanakal, Anubhab Das, Rahul Badri, Pradip Kar, Susanta Banarjee, Samarendra Maji

**Affiliations:** a Department of Chemistry, Faculty of Engineering and Technology, SRM Institute of Science and Technology (SRMIST) Kattankulathur Tamil Nadu-603203 India samarenr@srmist.edu.in; b Materials Science Centre, Indian Institute of Technology Kharagpur-721302 India; c Department of Chemistry, Birla Institute of Technology Mesra Ranchi Jharkhand-835215 India

## Abstract

This study presents the design of a sustainable, triazine-containing, polyurethane-based magnetic catalyst (Fe_3_O_4_@PUN-Pd) that exhibits better catalytic activity and desirable reusability for the efficient reduction of nitroarenes. Uniform polyurethane (PUN) microspheres were prepared by surfactant-free precipitation polymerization of toluene diisocyanate (TDI), the triazine-based polyol 3-THA and subsequently decorated with magnetic nanoparticles (MNPs) and palladium (Pd) to form magnetically separable Fe_3_O_4_@PUN-Pd catalysts. Comprehensive characterisation using FT-IR, XRD, HRSEM, HRTEM, EDX, TGA, DLS, UV-vis, ICP-OES and XPS confirmed the molecular interactions, structural integrity, thermal stability, and uniform dispersion of Pd NPs. HRSEM confirmed PUN's spherical shape, while XPS determined the surface-bound Pd's oxidation state. The resulting catalyst offers a sustainable platform with substantial recovery and reuse potential. The synthesized catalysts were evaluated for their hydrogenation activity towards 4-nitrophenol (4-NP) in an aqueous medium. The catalytic efficiency varied depending on the method used to prepare the MNPs and the Pd NP loading. Among the prepared catalysts with different synthesis methods, Fe_3_O_4_@PUN-Pd(2) demonstrated the best performance in the aqueous-phase hydrogenation of 4-nitrophenol (4-NP), delivering a high rate constant of 0.430 min^−1^ and maintaining ∼95% conversion over 10 consecutive cycles, possessing high stability and magnetic recoverability. The catalyst showed good activity and stability in water compared to organic solvents *viz.*, methanol, ethanol, and acetonitrile, underscoring its green applicability. Additionally, the reduction of various hazardous nitroarenes, such as 4-nitroaniline (4-NA), 2-nitroaniline (2-NA), nitrobenzene (NB) and 2-nitrotoluene (2-NT), was also investigated in aqueous medium. The catalyst showed potent stability and good catalytic performance in water, a green solvent, compared to methanol, ethanol, and acetonitrile.

## Abstract 2

Development of magnetically recyclable triazine-based polyurethane microspheres adorned with Pd NPs using a sustainable approach for the catalytic reduction of nitroarenes.

## Graphical abstract

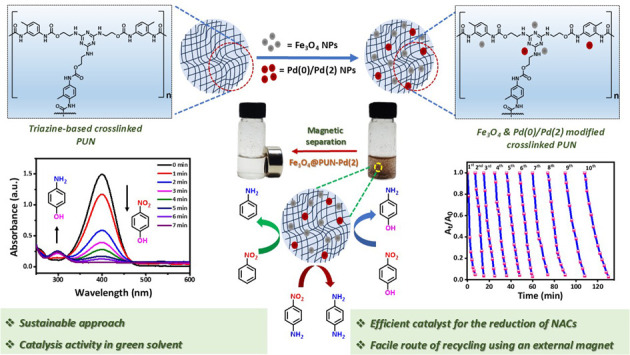

## 1 Introduction

The removal of organic pollutants from the environment, along with their increasing utilisation in agriculture and industries, has become a major concern for environmental scientists.^1^ Among these pollutants, nitroarenes are particularly harmful. They are generated by various industries, including dyes, pesticides, printing, cosmetics, pharmaceuticals, leather, explosives, and textiles.^2,3^ Nitroarenes are classified as hazardous pollutants due to their non-biodegradable and carcinogenic nature, which can have significant adverse effects on the environment.^4^ According to the Clean Water Act, the United States Environmental Protection Agency (US-EPA) has identified nitroarenes as major pollutants due to their potential carcinogenicity and toxicity. The agency recommends maintaining their concentration in natural water bodies at levels as low as 10.0 ng mL^−1^.^5^ Nitroarenes serve as economical and abundant precursors for the synthesis of many synthetic compounds, which can be readily transformed into aromatic amines, offering environment friendly alternatives through catalytic processes.^6^ For example, 4-nitrophenol (4-NP) is a common organic pollutant that is harmful to both human and aquatic life. Its stable chemical structure makes it difficult to eradicate from wastewater. Therefore, the chemical reduction method that converts 4-NP into 4-aminophenol (4-AP) using hydrogen sources such as lithium aluminium hydride, hydrazine hydrate, and sodium borohydride (NaBH_4_) as a reductant, along with a suitable catalyst, has gained significant interest.^7,8^ Furthermore, 4-AP is an important industrial raw material and an essential intermediate primarily used in the production of agrochemicals, dyes, corrosion inhibitors, antipyretic drugs and analgesics.^9^ Hence, the researchers are currently focusing on the development of highly effective, recoverable, and reusable catalysts for ecologically sustainable industrial applications.^10^

Over the past few decades, ultrafine noble metal nanoparticles (NPs) such as platinum (Pt), palladium (Pd), gold (Au), and silver (Ag) have been extensively studied due to their high surface-to-volume ratio, catalytic activity, superior mechanical, chemical, and thermal stability.^11,12^ Palladium nanoparticles (Pd NPs) have been recognised as a promising choice for catalysis due to their exceptional catalytic activity.^13,14^ Pd NP-based catalysts have been extensively used in heterogeneous catalysis for various reactions, including coupling,^15^ hydrogenation,^16^ and reduction reactions,^17^ because of their superior performance. The catalytic performance of Pd NPs is profoundly impacted by the active atoms on their surface, which are associated with their surface properties.^18^ Smaller Pd NPs tend to exhibit better catalytic properties due to their enhanced surface-to-volume ratio.^19^ However, a major drawback of ultrafine NPs is their tendency to agglomerate due to high surface energy and low stability. This agglomeration reduces the number of available active surface atoms, resulting in a significant loss of catalytic activity. While recycling and reusing prominent catalysts are essential for cost-effective and sustainable processes, their separation remains a challenging task.^20^ Traditional methods, such as centrifugation or filtration, may not be suitable for recovering these catalysts.^21^

To address these issues, active metal NPs have been embedded into solid supports, such as metal oxides,^22^ carbon materials,^23^ various types of polymers, including synthetic^24,25^ and natural biopolymers,^26^ and composite materials.^27^ The emergence of magnetic substrate-supported catalysts is of growing importance, owing to their efficient magnetic recoverability and minimal catalytic loss, which are critical for applications ranging from magnetic resonance imaging to targeted drug delivery.^28,29^ In heterogeneous catalytic systems, Fe_3_O_4_ magnetic nanoparticles (MNPs) are mainly considered as effective magnetically recoverable components. Their innate superparamagnetic characteristic enables quick and uncomplicated separation of the catalyst from the reaction media using an external magnetic field, reducing catalyst loss and simplifying reuse without the requirement of filtration or centrifugation. Fe_3_O_4_ NPs are a perfect magnetic core material because of their stability, biocompatibility, affordability, and ease of production, even if they are not usually used as an active catalyst since iron may interfere with redox conversions.^20,30,31^ When Fe_3_O_4_ NPs are embedded into a catalytically inert surface, such as polymers, silica, or carbon, they offer a robust magnetic backbone that supports the active catalytic species while retaining structural integrity. Among the above-mentioned supports, polyurethane (PUN) is a widely utilised synthetic polymer produced by the reaction of isocyanates with polyols, generating a cross-linked polymer network.^32^ Its excellent mechanical strength, chemical stability, and structural tunability support its broad applications in elastomers, coatings, and adhesives, while its inherent biocompatibility and biodegradability enable diverse biomedical uses, particularly in soft-tissue engineering.^33^ In particular, the biodegradation behaviour of PUN is strongly influenced by molecular orientation, crystallinity, degree of cross-linking, and the nature of chemical groups within the polymer chains, which collectively govern its accessibility to enzymatic degradation systems. In a related study, Sultan *et al.* reported the synthesis of cross-linked polyurethane foam (CPUF) and demonstrated its effectiveness in the catalytic reduction of methylene blue, exhibiting first-order kinetic behaviour.^34^ Khan *et al.* demonstrated a simple method for preparing MNPs coated with chitosan (CH) on a commercially available PUN sponge, achieving up to 90% conversion over three cycles.^35^ Yang *et al.* have prepared composite microspheres, *i.e.*, PUN-supported Pd (Pd@PUN), through precipitation polymerisation and modified with Pd NPs on the surface of the polymer.^36^ Jin *et al.* synthesised gold nanoparticles (AuNPs) on a polyurethane sponge catalyst using a one-step, *in situ* process, effectively reducing 4-NP and 2-nitroaniline (2-NA) while enabling recycling for up to five cycles.^37^ Notably, the integration of an *s*-triazine unit into the PUN backbone significantly improves its thermal, mechanical, and chemical stability, while also enhancing its overall processability.^38,39^ Triazine-based PUNs with high nitrogen and oxygen donor atoms facilitate the immobilisation of Pd NPs. Furthermore, incorporating a heterocyclic component such as a *s*-triazine unit into the design of PUN significantly influences its physicochemical and thermal characteristics, as well as its processability. For instance, Dey and colleagues synthesised Pd(0) NPs immobilised on triazine-based porous polyurethane (TPU-Pd) for the Suzuki–Miyaura cross-coupling reaction, allowing for recycling up to four cycles with a gradual loss of activity.^40^

To overcome the challenges of recyclability, in this study, we report a simple and cost-effective synthesis of magnetically separable triazine-based PUN microspheres that can be used as reusable heterogeneous catalysts. To the best of our knowledge, this is the first instance of a catalyst being designed for nitroaromatic reduction employing the triazine ring in the PUN matrix. The PUN microspheres were initially synthesised through precipitation polymerisation using toluene diisocyanate (TDI) and a triazine-based polyhydric alcohol (3-THA) in acetonitrile. In one approach, PUN-supported Fe_3_O_4_ NPs were prepared by loading the NPs onto the surface of the PUN microspheres, followed by modification with palladium(ii) acetate [Pd(OAc)_2_]. In another method, Fe_3_O_4_@Pd NPs were prepared and then modified with PUN microspheres. Comprehensive analysis using FT-IR, XRD, HRSEM, EDX, HRTEM, TGA, and XPS validated the structural, morphological, elemental, and thermal properties of the materials. The reduction of 4-NP was used to assess the catalytic performance, which was then expanded to additional hazardous nitroarenes. It showed good stability, potent activity, and recyclability in water, a green reaction medium, outperforming organic solvents.

## 2 Experimental section

### 2.1 Materials

2,4,6-Trichloro-1,3,5-triazine was purchased from Sigma-Aldrich. Palladium(ii) acetate (extrapure, 99%), ferric chloride hexahydrate (FeCl_3_·6H_2_O, AR, ACS, 97%), ferrous chloride tetrahydrate (FeCl_2_·4H_2_O, 98%), 25% aqueous ammonia, 2-aminoethanol (AR, ACS, 99%), sodium hydroxide, 4-nitrophenol (4-NP), 4-nitroaniline (4-NA), 2-nitroaniline (2-NA), nitrobenzene (NB), 2-nitrotoluene (2-NT), triethylamine (TEA), sodium borohydride (NaBH_4_, AR, ACS), sodium sulphate (dried), disodium ethylenediaminetetraacetate dihydrate (EDTA) (ACS, 99%) and isopropyl alcohol (IPA) (ACS, 99.5%) were purchased from SRL Pvt. Ltd, Mumbai, India. 1,4-Dioxane (AR, 99.5%), acetone (ACS, 99.5%), phenolphthalein (AR, 99%), and acetonitrile (HPLC & UV spectroscopy grade, 99.9%) were also obtained from SRL Pvt. Ltd. Toluene diisocyanate (2,4-, ∼80%; 2,6-, ∼20% TDI) was procured from TCI Chemicals Pvt. Ltd, India. Ethanol was purchased from Zenith Biochemical Industries Pvt. Ltd, Maharashtra, India. *p*-Benzoquinone (PBQ) was purchased from Research-Lab Fine Chem Industries, Mumbai, India. Ethyl acetate (EA) was obtained from Avantor Performance Materials India Ltd, Maharashtra, India. Milli-Q water with a resistivity higher than 18 MΩ.cm was utilised for every experiment.

### 2.2 Characterization

The synthesized compounds 3-THA, PUN, and Fe_3_O_4_@PUN-Pd(0)/Pd(2), Fe_3_O_4_@Pd(0)/Pd(2)-PUN microspheres were characterized by various methods such as Fourier transform infrared (FT-IR) spectroscopy, X-ray diffraction (XRD), nuclear magnetic resonance (NMR), high-resolution scanning electron microscopy with energy-dispersive X-ray spectroscopy (HRSEM-EDX), high-resolution transmission electron microscopy (HRTEM), dynamic light scattering (DLS), thermogravimetric analysis (TGA) and UV-vis spectroscopy. To examine the presence of different functional groups, FTIR studies were performed utilising a SHIMADZU IRTracer-100, ranging from 4000 to 400 cm^−1^. The XRD patterns were collected using a BRUKER USA D8 ADVANCE, Davinci and monochromatic Cu Kα radiation (1.5418 Å). A Bruker Avance 500 MHz was used to record the ^1^H-NMR spectrum of 3-THA at room temperature in dimethyl sulfoxide-*d*_6_ (DMSO-*d*_6_). The chemical shifts are given in parts per million (*δ*), relative to DMSO-*d*_6_ at 2.5 ppm. LC-MS analysis was performed on an LC-MS 2020 system equipped with an LC-10ADVP binary pump (Shimadzu, Japan). HRTEM was performed using a JEM-2100 Plus, JEOL Japan, with an accelerating voltage of 200 kV. An Apreo S (Thermo Scientific, USA) HRSEM-EDX with an accelerating voltage of 20 kV was used to record the surface morphology and elemental compositions. The particle size and zeta potential of the microspheres were measured with an Malvern/Nano ZS-90 instrument. XPS spectra were recorded on a PHI VersaProbe III (Shimadzu, Japan) spectrometer equipped with an Al Ka (1486.6 eV) radiation source. TGA analysis was carried out at temperatures ranging from 30 °C to 700 °C at a scan rate of 10 °C min^−1^ under an air atmosphere using a DTG-60, Shimadzu, Japan. A high dispersion Prodigy Inductively Coupled Plasma-Optical Emission Spectroscopy (ICP-OES) from Teledyne Leeman Laboratories Inc. (Model: Sl. No. 0114, USA) was used to detect the precise amount of metal loading using Inorganic Ventures 10 ppm multi-element standard solutions in 3% HNO_3_.

### 2.3 Methods

#### 2.3.1 Synthesis of triazine-based polyhydric alcohol (3-THA)

**Scheme 1:**
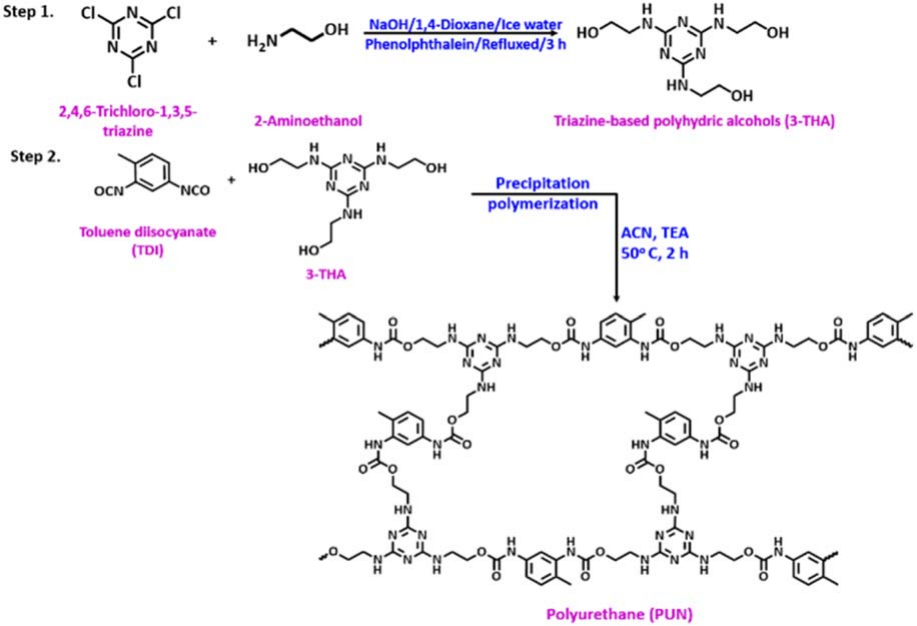
Schematic representation of the synthesis of polyurethane (PUN).

3-THA was synthesised according to the following procedure.^41^ First, 2,4,6-trichloro-1,3,5-triazine (18.44 g, 0.1 mol) was dissolved in 50 mL of 1,4-dioxane, which was poured into 60 mL of ice-cold water under vigorous stirring to form a slurry. 2-Aminoethanol (19.8 mL, 0.3 mol) was added to the slurry dropwise, and the reaction temperature was kept below 5 °C. A few drops of phenolphthalein were added to the reaction mixture, which was then gently heated to reflux and maintained for 3 hours. Sodium hydroxide (12 g, 0.3 mol) was dissolved in 30 mL of water and added dropwise to the reaction mixture during heating and reflux, taking care to avoid the appearance of phenolphthalein colouration (Scheme 1). The reaction mixture was cooled down to room temperature, the solvent was drained out, and the residue was dried. The residue was recrystallised using *n*-butanol, yielding the final product. The ^1^H NMR spectrum of the compound is shown in Fig. S1 (SI). ^1^H NMR (500 MHz, DMSO-*d*_6_): *δ* (ppm) 3.28 (m, 6H), 3.42 (m, 6H), 4.68 (br, 3H), 6.2–6.6 (t, 3H).

#### 2.3.2 Synthesis of PUN

PUN microspheres were prepared by adding acetonitrile (10 mL) and 3-THA (500 mg, 1.93 mmol) into a 50 mL reaction flask. The mixture was then sonicated for approximately 30 minutes. TDI (500.4 mg, 2.9 mmol) and TEA (39.02 µL) were added to the flask. 3-THA and TDI were added in such a way that the ratio of both functional groups would be equal (NCO/OH = 1). Argon gas was passed through the reaction for 30 minutes. After that, the mixture was placed in an oil bath at 50 °C and heated for 2 h. The reaction mixture was separated using a centrifuge, and the collected solid was washed three times with acetonitrile. It was then dried at 50 °C for 12 h to attain the PUN microspheres (Scheme 1).^36^

#### 2.3.3 Synthesis of magnetic nanoparticles (MNPs)

FeCl_3_·6H_2_O (10.8 g, 0.04 mol) and FeCl_2_·4H_2_O (3.9 g, 0.02 mol) were added to 200 mL of deionised water and stirred mechanically at 500 rpm under an argon atmosphere. After 30 minutes, 30 mL of 25 wt% NH_4_OH solution was added within 10–15 min. The obtained solution was then stirred for 1 h at room temperature, followed by heating at 85 °C for 1 h. To remove the excess ammonia, the temperature was increased to 110 °C for 1 h and then cooled to room temperature. With the help of a magnet, the black particles were collected, and the supernatant liquid was separated. The particles were rinsed with distilled water until the liquid after wash reached a pH between 7 and 8. Finally, the particles were dried in a vacuum oven at 50 °C until constant weight to get Fe_3_O_4_ NPs (MNPs).

#### 2.3.4 *In situ* preparation of Fe_3_O_4_@PUN NPs

In this method, Fe_3_O_4_@PUN NPs were prepared by a novel *in situ* method. First, PUN and Milli-Q water were mixed in a round-bottomed flask using a magnetic stirrer at 80 °C in an inert atmosphere. When the solution turned into a clear and homogeneous mixture, 12 mL of 25 wt% ammonia was slowly added to the mixture until a pH of 12 was obtained. Then, FeCl_2_·4H_2_O (1 g, 0.005 mol) and FeCl_3_·6H_2_O (2.5 g, 0.009 mol) were dissolved in Milli-Q water and added dropwise into the flask. Dark colored particles were formed, and the mixture was blended for an additional 2 h at room temperature. Fe_3_O_4_@PUN NPs were then magnetically captured and frequently washed with acetone and distilled water. The final product was dried at 70 °C in a vacuum oven until it reached a constant weight.

#### 2.3.5 Preparation of Fe_3_O_4_@PUN-Pd(2) microspheres

First, an aqueous solution of palladium(ii) acetate, Pd(OAc)_2_ (3.3 wt%), was prepared in a 5 mL glass vial. Then, a mixture of Fe_3_O_4_@PUN microspheres (300 mg) in 10 mL of Milli-Q water was prepared in a 50 mL beaker. Later, both solutions were mixed and evenly distributed by ultrasonication for 10 minutes. Finally, the mixture was stirred for 2 hours at room temperature. The resultant Fe_3_O_4_@PUN-Pd(2) microspheres were recovered from the reaction mixture by centrifugation and washed with Milli-Q water and ethanol, followed by drying at room temperature (35 °C).

#### 2.3.6 Preparation of Fe_3_O_4_@PUN-Pd(0) microspheres

Fe_3_O_4_@PUN-Pd(0) microspheres were prepared as discussed in Section 2.3.5. along with the addition of NaBH_4_ (12 mg, 0.32 mmol) at the time of reaction.

#### 2.3.7 Preparation of Fe_3_O_4_@Pd(2)-PUN

To prepare Fe_3_O_4_@Pd(2)-PUN, Fe_3_O_4_@Pd(2) NPs were first synthesized. In this case, distilled water (5 mL) and MNPs (10 mg) were added to a 50 mL beaker, followed by ultrasonication for 2 min. Later, ethanol (5 mL) and an aqueous solution of Pd(OAc)_2_ (0.6 mL, 6 mg/0.6 mL) were added to the 50 mL beaker containing MNPs. After 2 h of stirring, the Fe_3_O_4_@Pd(2) NPs were collected by centrifugation and washed thoroughly with Milli-Q water, followed by drying at 50 °C to obtain the Fe_3_O_4_@Pd(2) NPs. Finally, Fe_3_O_4_@Pd(2)-PUN was synthesised by mixing the obtained Fe_3_O_4_@Pd(2) NPs with PUN (25 mg) in ACN (5 mL), followed by stirring for 2 h. Solid Fe_3_O_4_@Pd(2)-PUN microspheres were recovered by centrifuging, washing with Milli-Q water and drying at room temperature.

#### 2.3.8 Preparation of Fe_3_O_4_@Pd(0)-PUN

Fe_3_O_4_@Pd(0)-PUN microspheres were synthesized by using a similar method as discussed earlier in Section 2.3.7, where first Fe_3_O_4_@Pd(0) NPs were prepared in the presence of NaBH_4_ (7 mg, 0.185 mmol) and an aqueous solution of Pd(OAc)_2_ (0.6 mL, 6 mg/0.6 mL). Finally, the obtained Fe_3_O_4_@Pd(0) NPs were added to PUN (25 mg) microspheres in acetonitrile (5 mL) kept in a 50 mL beaker and stirred for 2 h. After centrifugation, the solid Fe_3_O_4_@Pd(0)-PUN microspheres were collected and dried to remove the solvent.

## 3 Results and discussion

### 3.1 Synthesis of 3-THA, PUN, Fe_3_O_4_@PUN, Fe_3_O_4_@PUN-Pd(0), Fe_3_O_4_@PUN-Pd(2), Fe_3_O_4_@Pd(0)-PUN and Fe_3_O_4_@Pd(2)-PUN

3-THA was synthesized by reacting 2,4,6-trichloro-1,3,5-triazine and 2-aminoethanol in 1,4-dioxane solvent with a few drops of phenolphthalein solution and NaOH, as shown in Scheme 1, as reported earlier.^41^ This is a typical S_N_2 reaction, where the –NH_2_ group in 2-aminoethanol acts as a nucleophile and attacks the C, attaching to the Cl in the triazine compound to generate HCl as a byproduct. NaOH was added to the reaction mixture to remove HCl. However, excess NaOH can deprotonate the –OH group in 3-THA; therefore, phenolphthalein was added to maintain a constant NaOH concentration. The structural confirmation of the product was done by ^1^H NMR spectroscopy, as shown in Fig. S1, SI. Methylene protons attached to the –OH group display a peak at *δ* 3.28 ppm. Whereas the signal at *δ* 3.42 ppm was due to –CH_2_ protons attached to the –NH group. The peak at *δ* 4.68 ppm corresponds to the hydroxyl group, and the peak at *δ* 6.2–6.6 ppm refers to secondary amine protons in triazine.

PUN was synthesized through the reaction of TDI and 3-THA. The bifunctional TDI with two isocyanate groups (–NCO) reacts with the hydroxyl (–OH) of 3-THA. The trifunctional THA polymerizes with bifunctional TDI in an A_3_ + B_2_ fashion and leads to the formation of crosslinked PUN and precipitates out from the polymerization medium. Electrostatic interaction between urethane bonds and electron-rich terminal triazine rings containing polar substituents enhances the compatibility of hard and soft segments of the PUN microspheres.

Subsequently, triazine-based PUN microspheres were modified by MNPs, giving Fe_3_O_4_@PUN, then Pd NPs were immobilised onto them to form Fe_3_O_4_@PUN-Pd(2). The modification of Pd by PUN microspheres hinders the aggregation of Pd NPs. Triazine-containing PUN acts as a good adsorbent for MNPs and Pd NPs. The N and O atoms of PUN coordinate with Pd.^36^ The triazine ring in PUN functions as a Lewis base to donate electron pairs to the Lewis acidic metal centre and operates as a ligand where nitrogen-rich atoms are the main locations for coordination. The reducing agent NaBH_4_ was added to synthesise the Fe_3_O_4_@PUN-Pd(0) catalyst, where Pd(2) was reduced to Pd(0). Whereas, in another method, MNPs were first modified with Pd NPs, followed by their incorporation on the surface of PUN to form Fe_3_O_4_@Pd(2)-PUN. Similarly, Fe_3_O_4_@Pd(0)-PUN was prepared in the presence of the reducing agent NaBH_4_.

### 3.2 Characterization of 3-THA, PUN, Fe_3_O_4_@PUN, Fe_3_O_4_@PUN-Pd(0), Fe_3_O_4_@PUN-Pd(2), Fe_3_O_4_@Pd(0)-PUN and Fe_3_O_4_@Pd(2)-PUN

#### 3.2.1 FT-IR analysis

Fourier transform infrared spectra of the prepared compounds are shown in Fig. S2, SI. In the case of PUN spectrum (Fig. S2a, SI), the peak at 3332 cm^−1^ is related to the stretching vibrational peak of the amine (N–H) group, and the peak at 1700 cm^−1^ indicates the hydrogen-bonded urethane carbonyl (C=O) stretching band. The presence of N–H and C=O bonds denotes the formation of urethane bonds. The peak at 2270–2300 cm^−1^ has almost vanished, indicating that the isocyanate (–N=C=O) group has polymerised. The absorption peak at 2944 cm^−1^ is due to the stretching of the –CH_2_ group. The bending vibrational peak of the N–H bond has appeared at 1528 cm^−1^. The stretching vibrational peak at 1236 cm^−1^ is assigned to C=O connected to the N–H bond.^42^ The absorption band at 1041 cm^−1^ is attributed to the C–O stretching band (Fig. S2a, SI).

In the case of Fe_3_O_4_@PUN, all the peaks corresponding to PUN are present; in addition, a characteristic peak of the Fe–O stretching band is observed at 571 cm^−1^, indicating the coordination of Fe_3_O_4_ NPs with the O atom of PUN (Fig. S2b, SI).^43,44^ The shift of C=O and C–O to a lower frequency is due to the interaction of oxygen with NH of the urethane bond to form complexes. This was perhaps due to hydrogen bond formation between PUN and MNPs, this H-bonding improves the adhesion between the NPs and the polymer matrix.^45^ The spectra of Fe_3_O_4_@PUN-Pd(0), Fe_3_O_4_@PUN-Pd(2) (Fig. S2c and S2d, SI) exhibit the same peak as present in PUN and Fe_3_O_4_@PUN. The absorption spectra of Fe_3_O_4_@Pd(0) and Fe_3_O_4_@Pd(2) are shown in Fig. S2e and S2f, SI. The peak at 3382 cm^−1^ demonstrates the existence of the O–H stretching band on the surface of Fe_3_O_4_ NPs. All the characteristic stretching bands of the PUN are present in the FTIR spectrum of Fe_3_O_4_@Pd(0)-PUN and Fe_3_O_4_@Pd(2)-PUN, supporting the successful modification of PUN (Fig. S2g and S2h, SI). The triazine ring plays a major role in Pd encapsulation. The lone pair of the –NH– group attached to the triazine ring coordinated with Pd. In addition, N of triazine can bind with Pd. Finally, a strong ligand-to-metal charge transfer transition took place from p_π_ orbitals of the donor atoms to the d orbitals of Pd.^46^

#### 3.2.2 XRD analysis

**Fig. 1:**
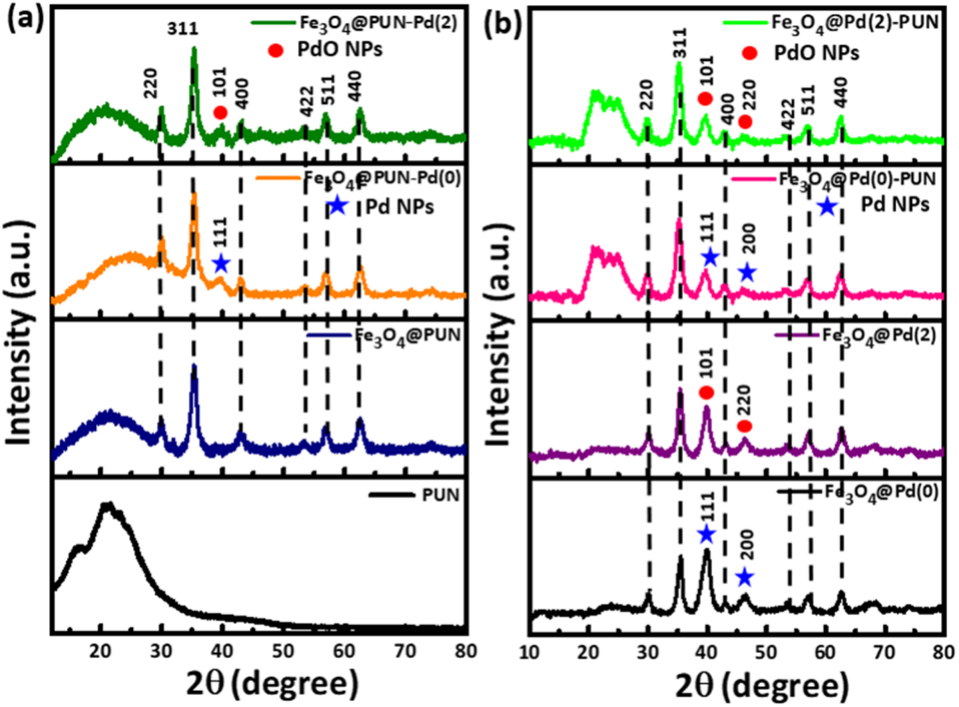
(a) XRD patterns of PUN, Fe_3_O_4_@PUN, Fe_3_O_4_@PUN-Pd(0), and Fe_3_O_4_@PUN-Pd(2). (b) XRD patterns of Fe_3_O_4_@Pd(0), Fe_3_O_4_@Pd(2), Fe_3_O_4_@Pd(0)-PUN, and Fe_3_O_4_@Pd(2)-PUN.

The XRD patterns of PUN, Fe_3_O_4_@PUN, Fe_3_O_4_@PUN-Pd(0), Fe_3_O_4_@PUN-Pd(2), Fe_3_O_4_@Pd(0), Fe_3_O_4_@Pd(2), Fe_3_O_4_@Pd(0)-PUN, Fe_3_O_4_@Pd(2)-PUN are shown in Fig. 1. The characteristic broad peak at 21.43° is ascribed to the amorphous peak of PUN, while strong diffraction peaks of Fe_3_O_4_@PUN observed at angles (2*θ*) 30.2, 35.42, 43.19, 53.57, 57.2, and 62.63 are assigned to (220), (311), (400), (422), (511), and (440) Miller indices of magnetite Fe_3_O_4_, respectively (JCPDS 75-1610).^47,48^ The crystallographic planes (111) and (200), corresponding to Bragg's reflection at 2*θ* values of 39.6° and 46.17°, confirm the face-centred cubic structure of Pd NPs in the case of Fe_3_O_4_@Pd(0) and Fe_3_O_4_@Pd(0)-PUN, respectively.^49^ However, Fe_3_O_4_@Pd(2) and Fe_3_O_4_@Pd(2)-PUN contain diffraction lines at (101) and (220) of the PdO phase,^50,51^ along with the robust pattern of Fe_3_O_4_ and a feeble peak of PUN in the case of the polymer-modified catalyst. Fe_3_O_4_@PUN-Pd(0) and Fe_3_O_4_@Pd(2)-PUN show the planes (111) and (101), attributed to the presence of Pd NPs and PdO NPs in small amounts.^50,51^ The above-mentioned diffraction peaks are observed in all the cases where Fe_3_O_4_, Pd and PdO NPs are present, with slight variation in the 2*θ* (degree) value. In Fig. 1b, there is no broad peak present in Fe_3_O_4_@Pd(0) and Fe_3_O_4_@Pd(2) due to the absence of PUN. These results support the effective incorporation of Pd and MNPs on the PUN microspheres. The Debye–Scherrer equation was utilized to find the crystallite size of the prepared catalyst expressed as eqn (1).^10^1
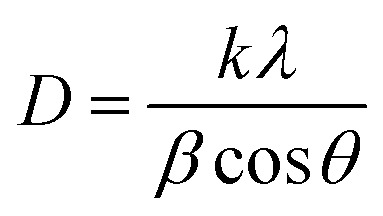
where *D* is the crystallite size, *k* is the Scherrer constant (0.89), *β* is the full width at half-maximum (FWHM) of diffraction peaks in radians, and *θ* is the Bragg's angle. The average crystallite size of Fe_3_O_4_@PUN, Fe_3_O_4_@PUN-Pd(0), Fe_3_O_4_@PUN-Pd(2), Fe_3_O_4_@Pd(0), Fe_3_O_4_@Pd(2), Fe_3_O_4_@Pd(0)-PUN and Fe_3_O_4_@Pd(2)-PUN corresponds to 6.13 nm, 7.52 nm, 7.09 nm, 7.11 nm, 7.37 nm, 6.60 nm and 6.61 nm, respectively. There is a decrease in the size after incorporating the polymer into the NPs due to the steric repulsion, which helps to prevent aggregation, stabilise particles and maintain a smaller average particle size.

#### 3.2.3 SEM analysis

**Fig. 2:**
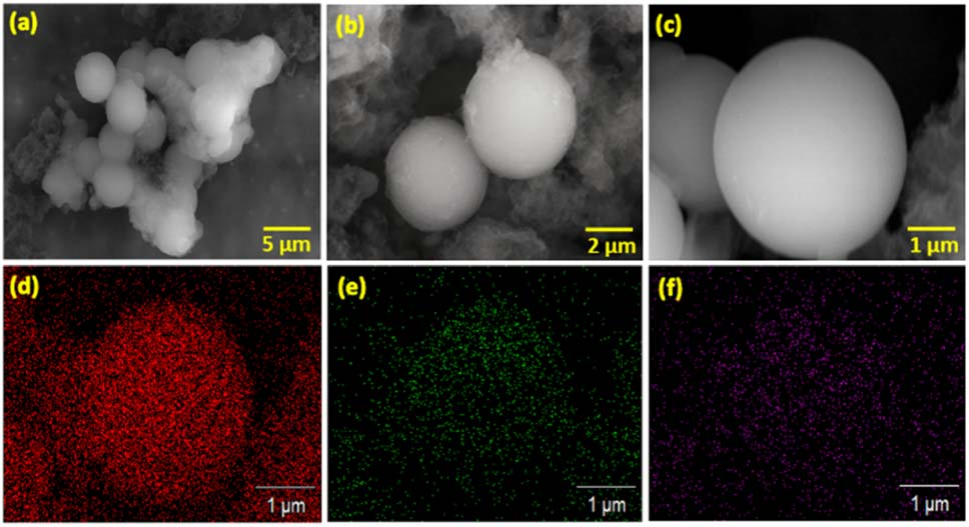
SEM images of PUN microspheres (a, b and c) at different magnifications. Elemental mapping images of (d) carbon, (e) nitrogen, and (f) oxygen.

**Fig. 3:**
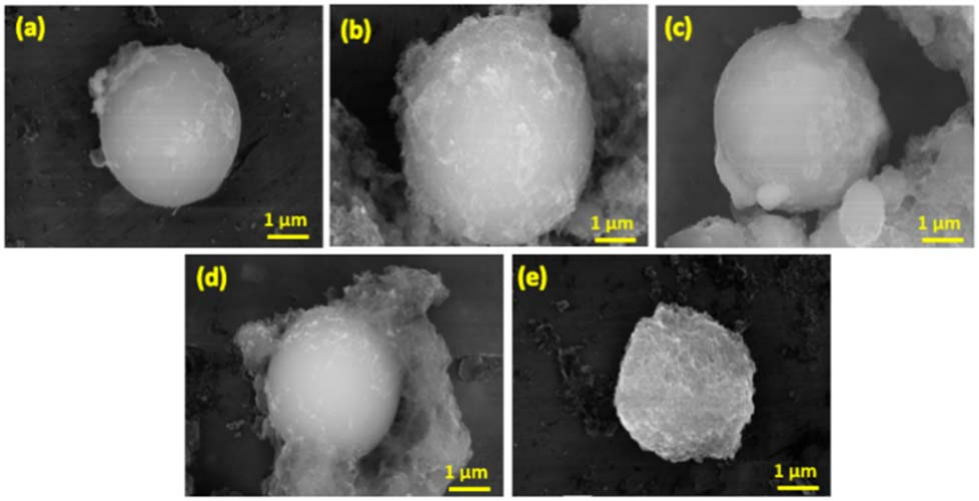
SEM images of (a) Fe_3_O_4_@PUN, (b) Fe_3_O_4_@PUN-Pd(0), (c) Fe_3_O_4_@PUN-Pd(2), (d) Fe_3_O_4_@Pd(0)-PUN, and (e) Fe_3_O_4_@Pd(2)-PUN.

SEM images of microspheres and the catalysts are shown in Fig. 2 and 3, respectively, which provide morphology and uniformity in the microspheres. According to the SEM images, the microspheres display spherical morphology with an average diameter of 2.97 µm for PUN. Energy-dispersive X-ray spectroscopy (EDX) spectra (Fig. S3, SI) and elemental mapping images (Fig. 2d–f) aid in understanding the formation of PUN microspheres with the elements present, *viz.* carbon, nitrogen and oxygen. After modifying PUN microspheres with Fe_3_O_4_ and Pd NPs, there is no significant difference in the internal structure of PUN microspheres. The average diameter of Fe_3_O_4_@PUN, Fe_3_O_4_@PUN-Pd(0), Fe_3_O_4_@PUN-Pd(2), Fe_3_O_4_@Pd(0)-PUN, and Fe_3_O_4_@Pd(2)-PUN is as follows: 2.85 µm, 3.23 µm, 3.76 µm, 3.57 µm, and 2.22 µm, respectively. After being modified with Pd, the size of the microspheres increased because interaction between the triazine unit and metal NPs can serve as a linker between polymeric chains, creating a more linked network and increasing the material's total size. The EDX mapping images of Fe_3_O_4_@PUN, Fe_3_O_4_@PUN-Pd(0), and Fe_3_O_4_@PUN-Pd(2) are shown in Fig. S4 (SI), which confirm the presence of Pd and MNPs on the surface of PUN microspheres after modification.

#### 3.2.4 TEM analysis

**Fig. 4:**
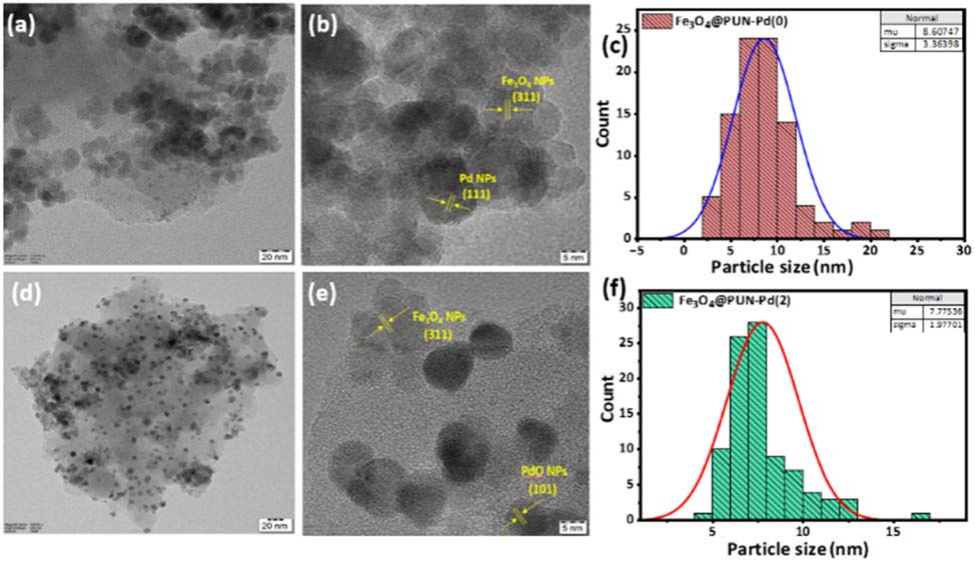
TEM images of Fe_3_O_4_@PUN-Pd(0) (a and b) and Fe_3_O_4_@PUN-Pd(2) (d and e), and their respective particle size distribution curve of MNP@Pd NPs (c and f).

The structure and average particle size were further inspected by HRTEM. Fig. 4 demonstrates the inclusion of MNPs and Pd NPs [Pd(0) and Pd(2)] on the PUN surface. The metal NPs (dark circles) were anchored almost uniformly to the semi-transparent polymer, which indicates the strong interaction between the coordinated centre (triazine, O) of the polymer and the metal NPs. Some aggregation is observed from the HRTEM images of Fe_3_O_4_@PUN-Pd(0) and Fe_3_O_4_@PUN-Pd(2) owing to the magneto-dipole interactions of MNPs. HRTEM images of Fe_3_O_4_@PUN-Pd(0) and Fe_3_O_4_@PUN-Pd(2) show distinct lattice fringes with a *d* spacing of 0.25 nm corresponding to the (311) lattice plane of Fe_3_O_4_ NPs.^52^ Inverse fast Fourier transform images ascertained the lattice fringes of Pd and PdO NPs with an interplanar distance of ∼0.224 nm and 0.263 nm to (111) and (101) lattice planes, respectively (Fig. 4b and e).^53,54^ The histograms of Fe_3_O_4_@PUN-Pd(0) and Fe_3_O_4_@PUN-Pd(2) show average particle sizes of MNPs with Pd(0) and Pd(2) of 8.06 ± 3.36 nm and 7.77 ± 1.97 nm, respectively (Fig. 4c and f). The average crystallite size and lattice planes obtained from the XRD pattern are in agreement with the HRTEM results. Fig. S5b and S5d, SI show the bright circular rings in the selected area, which refer to electron diffraction (SAED) patterns of Fe_3_O_4_, Pd and PdO NPs with hkl planes. The HRTEM images of Fe_3_O_4_@Pd(0)-PUN (Fig. S6a–S6c, SI) and Fe_3_O_4_@Pd(2)-PUN (Fig. S6d–S6f, SI) represent the fabrication of Fe_3_O_4_@Pd NPs on the PUN surface at 20 nm, 10 nm and 2 nm resolutions, respectively. The occurrence of C, N, O, Fe and Pd elements was validated by energy-dispersive X-ray (EDX) spectra (Fig. S7, SI), which proved the immobilization of Pd and Fe_3_O_4_ NPs onto the PUN surface. Fe_3_O_4_@PUN-Pd(0) contains 8.85 atomic % of Fe and 0.1 atomic % of Pd, whereas Fe_3_O_4_@PUN-Pd(2) contains 5.77 atomic % of Fe and 0.44 atomic % of Pd.

#### 3.2.5 DLS study

DLS is a technique used to validate the particle size, stability and polydispersity index of the particles. The average particle size was calculated and mentioned in Table S1. The particle size of PUN is 3023 nm. Upon modification with Fe_3_O_4_ (Fe_3_O_4_@PUN) and Pd(0) [Fe_3_O_4_@PUN-Pd(0)], the particle size increases (3286 and 3670 nm, respectively). Again, the large size of Fe_3_O_4_@PUN-Pd(0) is due to the aggregation of the active Pd(0) species.^55^ However, in the case of Fe_3_O_4_@PUN-Pd(2), the possible attraction between Pd(2) species and PUN surface reduces the size (1712 nm). A similar trend was noticed in the case of Fe_3_O_4_@Pd(0)-PUN (2354 nm) and Fe_3_O_4_@Pd(2)-PUN (1831 nm). The polydispersity index (PDI) value of all the samples was found to be around 0.10–0.47, which indicates that the particles are well dispersed in an aqueous medium. PUN with a nitrogen-rich triazine unit can easily coordinate with Fe_3_O_4_ and Pd NPs due to their strong donor properties. This interaction is mainly utilised to stabilise the NPs, preventing them from leaching and clogging together, to develop highly effective, reusable catalysts.

**Fig. 5:**
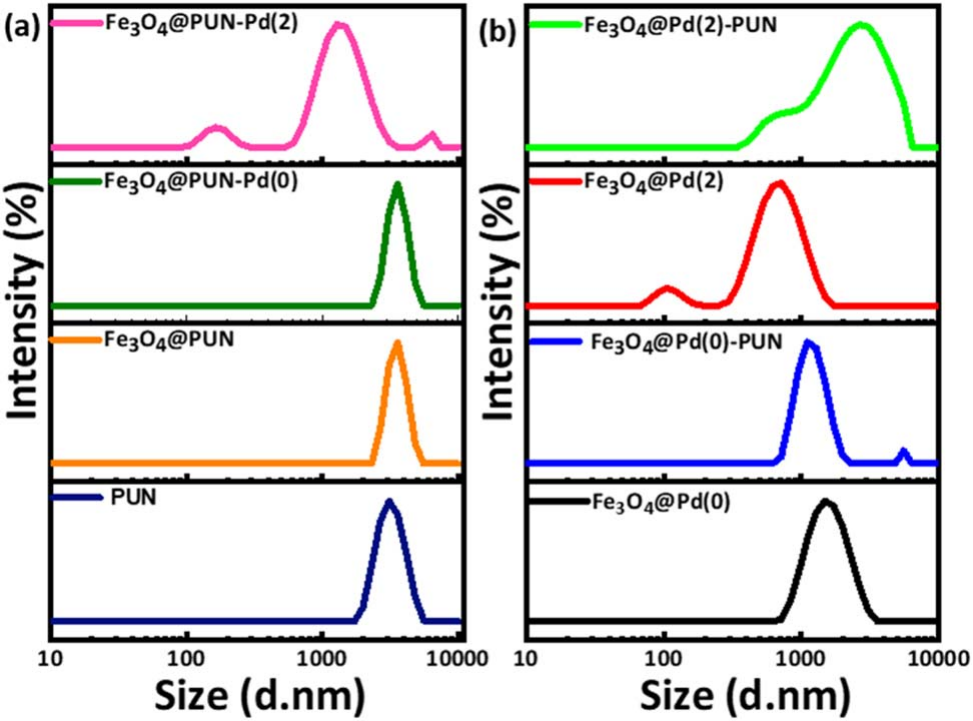
Size distribution of (a) PUN, Fe_3_O_4_@PUN, Fe_3_O_4_@PUN-Pd(0), and Fe_3_O_4_@PUN-Pd(2). (b) Fe_3_O_4_@Pd(0), Fe_3_O_4_@Pd(0)-PUN, Fe_3_O_4_@Pd(2), and Fe_3_O_4_@Pd(2)-PUN.

Zeta potential is considered an important factor in determining the stability of the NPs.^56,57^ The zeta potential of PUN, Fe_3_O_4_@PUN(0), Fe_3_O_4_@PUN-Pd(0) was found to be −5.70 mV, −5.25 mV, and −8.96 mV, respectively (Fig. 5). For other samples, the zeta potential is between 3 and 10 mV. In the case of PUN and Fe_3_O_4_@PUN, the negative value of zeta potential indicates the presence of negatively charged triazine on the surface. However, the introduction of a positively charged Pd(2) on the surface was reflected in a positive zeta potential. These findings validate the successful modification of PUN with MNPs and Pd NPs.

#### 3.2.6 TGA

**Fig. 6:**
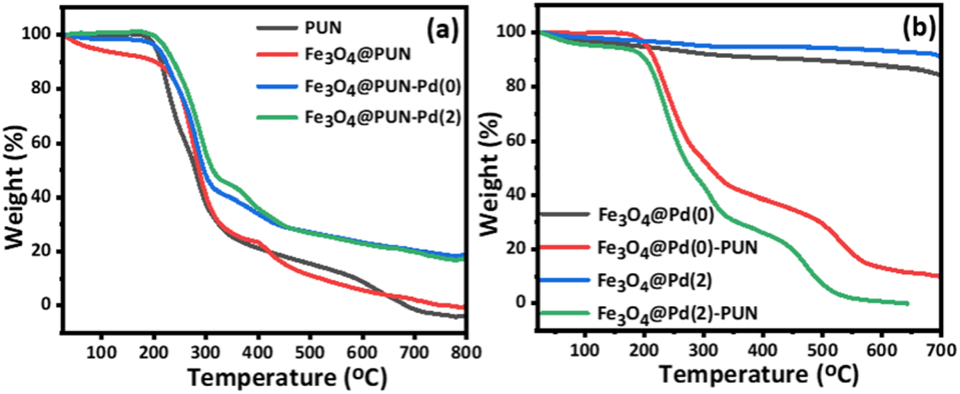
TGA plot of (a) PUN, Fe_3_O_4_@PUN, Fe_3_O_4_@PUN-Pd(0), and Fe_3_O_4_@PUN-Pd(2). TGA plots of (b) Fe_3_O_4_@Pd(0), Fe_3_O_4_@Pd(0)-PUN, Fe_3_O_4_@Pd(2), and Fe_3_O_4_@Pd(2)-PUN.

The thermal stability of the prepared catalyst was investigated by thermogravimetric analysis (TGA). Initially, PUN was stable up to 198 °C; however, 3% mass loss was observed due to the evaporation of physically absorbed water (Fig. 6a). PUN microspheres were instantly degraded after 198 °C and retarded at 316 °C, with a residual mass of 26.07%. This degradation is due to the decomposition of the urethane bond.^58,59^ Finally, at the decomposition temperature of around 610 °C, a weight residue of around 9% was obtained. This is due to the presence of a triazine unit in the PUN backbone, which increases the stability of PUN;^41^ in contrast, a non-triazine-containing PUN was found to be degraded completely at 450 °C.^36^

The thermal profile of PUN and Fe_3_O_4_@PUN was similar, with slight variation in degradation. In the degradation of Fe_3_O_4_@PUN in the first stage, from 35 to 221 °C, ∼10% weight loss was observed due to the presence of water content. The 2nd degradation appeared at 403 °C, which can be ascribed to the decomposition of polymeric units, with the residual mass of 23%. The char yield of PUN-modified MNPs (Fe_3_O_4_@PUN) was high compared to bare PUN. It states that the incorporation of MNPs into the polymer matrix prevents the thermal degradation of PUN. The thermal degradation of Fe_3_O_4_@PUN-Pd(0) and Fe_3_O_4_@PUN-Pd(2) was similar to each other with trace changes in temperature. An initial weight loss of 3% at 20–200 °C in the case of Fe_3_O_4_@PUN-Pd(0) was due to desorption of the water moiety, whereas there was no significant weight loss in Fe_3_O_4_@PUN-Pd(2). In the 2nd step of 200–320 °C, the weight residue was 43% for Fe_3_O_4_@PUN-Pd(0) and 47% for Fe_3_O_4_@PUN-Pd(2) due to the degradation of polymer chains on the surface of the microsphere. Interestingly, there was an increase in the stability of Fe_3_O_4_@PUN-Pd(0) and Fe_3_O_4_@PUN-Pd(2), after coordinating PUN with Fe_3_O_4_@Pd NPs, which can improve their thermal stability. NPs and the polymer matrix have a strong hydrogen bonding interaction between them, which hinders the movement of the polymeric chain and protects it from further degradation.^60^ The thermal degradation of both catalysts around ∼455 °C corresponds to the decomposition of the interlayer region of carbonates. The char yield was observed around 29%, which is greater than that of Fe_3_O_4_@PUN and PUN.

The thermal profiles of Fe_3_O_4_@Pd(0), Fe_3_O_4_@Pd(2), Fe_3_O_4_@Pd(0)-PUN and Fe_3_O_4_@Pd(2)-PUN are shown in Fig. 6b. The initial weight loss of 2% to 8% was noticed in all the samples before 200 °C, which was due to the evaporation of water content. The thermal degradation of Fe_3_O_4_@Pd(0) and Fe_3_O_4_@Pd(2) was witnessed at 360 °C and 344 °C, respectively, corresponding to the weight loss of 9% and 7%, respectively. The char yield was also high in both materials. The TGA curves of Fe_3_O_4_@Pd(0)-PUN and Fe_3_O_4_@Pd(2)-PUN exhibited weight residues of 42% and 39%, respectively, at 200–345 °C. This may be due to the degradation of the PUN chain grafted onto Fe_3_O_4_@Pd NPs. For both compounds, at 440–500 °C, there was a weight loss of 12%, and the slow degradation was observed at 530–570 °C due to depletion of carbon compounds. The microspheres synthesised in this work are comparatively more stable than previously reported microspheres, Pd@PUN.^36^ The degradation rate of Pd@PUN slowed down at 350 °C, whereas Fe_3_O_4_@PUN-Pd(2) decomposed at ∼500 °C, which shows the stability of the microspheres.

#### 3.2.7 XPS analysis

**Fig. 7:**
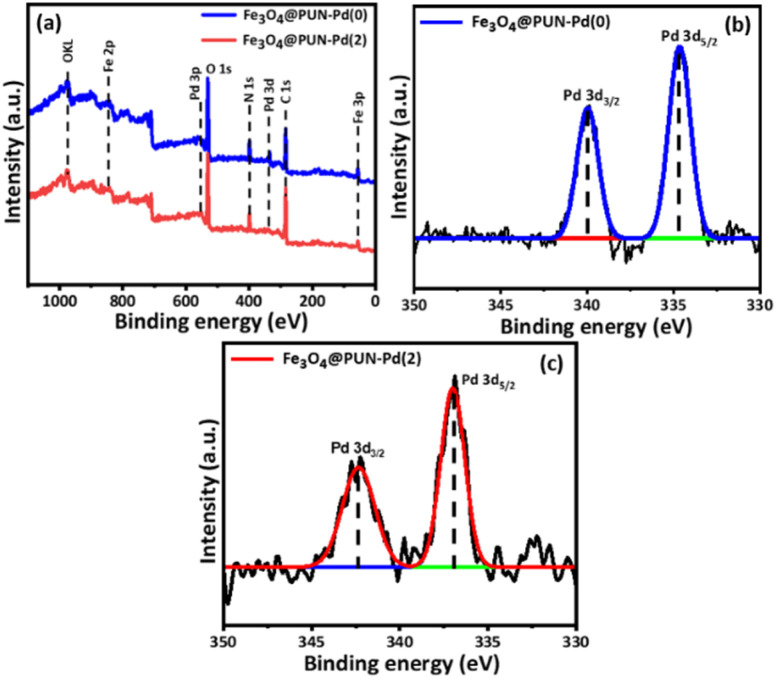
(a) XPS survey spectrum of Fe_3_O_4_@PUN-Pd(0) and Fe_3_O_4_@PUN-Pd(2). XPS spectra of (b) Pd(0) of Fe_3_O_4_@PUN-Pd(0) and (c) Pd(2) of Fe_3_O_4_@PUN-Pd(2).

XPS analysis was accomplished to elucidate the oxidation state of elements present in Fe_3_O_4_@PUN-Pd(0) and Fe_3_O_4_@PUN-Pd(2). Fig. 7a represents the survey spectra of Fe_3_O_4_@PUN-Pd(0) and Fe_3_O_4_@PUN-Pd(2), where Pd 3d, Fe 2p, O 1s, C 1s, and N 1s peaks were observed. In the XPS spectra of Fe_3_O_4_@PUN-Pd(0) (Fig. 7b), the peaks are observed at binding energies of ∼335 eV and ∼340 eV related to the spin–orbit splitting of Pd 3d_5/2_ and Pd 3d_3/2_ elements of Pd(0) state, respectively.^61^ This confirms that the Pd(0) NPs are successfully incorporated into the PUN. In Fig. S8a (SI), the presence of Fe_3_O_4_ NPs was confirmed by two signals around 710 eV and 724 eV, corresponding to Fe 2p_3/2_ and Fe 2p_1/2_, respectively.^62^ The deconvoluted spectra of O 1s imply the binding energies of 529 eV and 531 eV corresponding to Fe–O and C=O bonds, respectively (Fig. S8b, SI).^63^ The XPS spectrum of C 1s is deconvoluted into three major peaks at 283.2, ∼285 and 287.5 eV corresponding to C=C, C=O/C–N and O–C=O bonds, respectively (Fig. S8c, SI).^64^ In the XPS spectrum of Pd 3d in Fe_3_O_4_@PUN-Pd(2) (Fig. 7c), peaks appeared at binding energies of 337 and 342.3 eV, which are attributed to Pd 3d_5/2_ and Pd 3d_3/2_, respectively, which proves the presence of palladium metal in the Pd(2) state.^65^ The binding energy of Fe 2p, O 1s, and C 1s is similar to the above-mentioned value corresponding to Fe_3_O_4_@PUN-Pd(2) elements shown in Fig. S8d–S8f (SI). The presence of all these elements shows the successful incorporation of Fe_3_O_4_ and Pd NPs into the polymer microsphere. Furthermore, ICP-OES was used to analyse the Pd content in all the samples after acid digestion of the samples, and it was found to be 3.05 wt%, 0.92 wt%, 0.19 wt%, and 0.58 wt%, corresponding to Fe_3_O_4_@PUN-Pd(2), Fe_3_O_4_@PUN-Pd(0), Fe_3_O_4_@Pd(0)-PUN, and Fe_3_O_4_@Pd(2)-PUN, respectively.

### 3.3 Catalytic activity of the synthesized catalysts

**Fig. 8:**
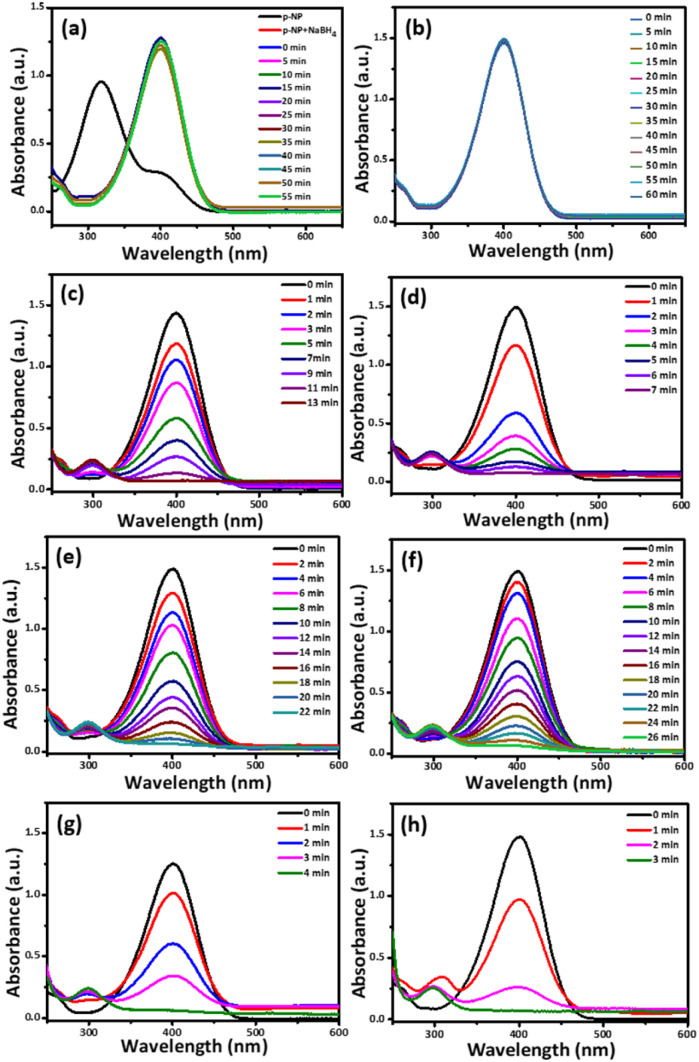
UV-visible spectra (a) before and after the addition of NaBH_4_, with the reaction carried out in the absence of catalyst and the reduction of 4-NP to 4-AP in the presence of (b) Fe_3_O_4_@PUN, (c) Fe_3_O_4_@PUN-Pd(0), (d) Fe_3_O_4_@PUN-Pd(2), (e) Fe_3_O_4_@Pd(0), (f) Fe_3_O_4_@Pd(2), (g) Fe_3_O_4_@Pd(0)-PUN, and (h) Fe_3_O_4_@Pd(2)-PUN.

The heterogeneous catalytic reduction of hydroxylated nitroarenes, such as nitrophenols, was considered one of the most harmful chemicals present in the water bodies for a long time.^66^ Hence, catalytic reduction of 4-NP to 4-AP played a vital role in a sustainable environment. The catalytic efficiency of the synthesised Pd-containing MNPs incorporated on PUN was investigated for the catalytic conversion of 4-NP into 4-AP, which was chosen as a model reaction in the presence of an excess of NaBH_4_. The hue of 4-NP transformed into an intense yellow colour, and the UV-vis absorption peak of 4-NP underwent a bathochromic shift from 317 nm to 400 nm, as shown in Fig. 8a, evidencing the generation of 4-nitrophenolate ions due to the deprotonation of the OH group of 4-NP in the presence of hydride originating from NaBH_4_.^48^ In the absence of catalyst, even when the potent reducing agent NaBH_4_ was added, the 4-nitrophenolate ions could not be reduced. This could be due to the high kinetic energy barrier between mutually repulsive anions of 4-NP and BH_4_^−^.^7^ Initially, we tried the 4-NP reduction in the presence of FeCl_2_, FeCl_3_ and Pd(OAc)_2_. Nevertheless, the reduction was time-consuming and without polymer support, and bare metallic compounds were leached into the reaction medium (Fig. S9a, SI). Later, the reduction of 4-NP was carried out with Fe_3_O_4_@PUN and Fe_3_O_4_@PUN-modified Pd.

With the addition of Fe_3_O_4_@PUN microspheres, the bright yellow coloured solution of 4-NP remained unchanged, and there is no reduction in the absorption peak even after 60 min, indicating the negligible catalytic activity (Fig. 8b). Upon addition of Pd modified Fe_3_O_4_@PUN, the absorbance signal of 4-nitrophenolate anions at 400 nm decreases at time intervals and generates another signal at 300 nm, interprets the conversion of 4-NP into 4-AP within 7–13 min. In the meantime, the 4-NP solution's intense yellow colour eventually faded to colourless. The concentration of BH_4_^−^ introduced to the system is higher than the concentration of 4-NP, and it is expected that the BH_4_^−^ concentration remains constant throughout the reaction. The catalytic properties of synthesised microspheres Fe_3_O_4_@PUN-Pd(0), Fe_3_O_4_@PUN-Pd(2), Fe_3_O_4_@Pd(0)-PUN and Fe_3_O_4_@Pd(2)-PUN were investigated by catalytic degradation of 4-NP, and the reduction occurred within 13, 7, 4 and 3 min, respectively (Fig. 8c, d, g and h). Of note, Fe_3_O_4_@Pd(0)-PUN and Fe_3_O_4_@Pd(2)-PUN show better catalytic performance (Fig. 8g and h); however, the stability and recyclability of the catalysts have been hindered in the system. Due to the lower Pd loading (ICP-OES results) in these catalysts, there was a decrease in the conversion rate after successive cycles. The effect of change in the Fe_3_O_4_@PUN-Pd(2) amount and NaBH_4_ concentration was also investigated (Fig. S9b and c, SI). When the catalyst loading was low, it took more time for the conversion of 4-NP, and when the amount of Fe_3_O_4_@PUN-Pd(2) was increased, the reduction was accelerated and completed within 7 minutes. In this model, BH_4_^−^ acts as a nucleophile, while 4-NP serves as an electrophile. The catalyst, Pd-NPs, functions as a promoter, facilitating electron transfer from the donor BH_4_^−^ to the acceptor 4-NP. Increasing the amount of catalyst results in more active sites, which accelerates the reduction rate.^67,68^ The amount of NaBH_4_ also affects reduction; lowering the concentration of NaBH_4_ (0.01 M) takes more time for reduction (22 min). As the concentration of NaBH_4_ was increased (0.05 M), the reduction time was shortened to 7 min. This indicates that an excessive amount of NaBH_4_ is required to gain higher catalytic performance for the faster reduction of 4-NP.^69^ Subsequently, in comparison between Fe_3_O_4_@PUN-Pd(2) and Fe_3_O_4_@PUN-Pd(0), the size of MNP@Pd is less for Fe_3_O_4_@PUN-Pd(2) (7.77 nm) compared to Fe_3_O_4_@PUN-Pd(0) (8.06 nm). Therefore, the smaller size exhibits more surface to volume ratio, hence demonstrating higher catalytic activity. Moreover, coordination plays a major role in the enhanced catalytic activity of Fe_3_O_4_@PUN-Pd(2). The Pd^2+^ centre of Fe_3_O_4_@PUN-Pd(2) is well known for making coordination with N and O in the polymer.^70^ Furthermore, Pd^2+^ has a vacant d-orbital (d^8^), which can take part in a strong coordination with an N-donor.^71^ As a result, strong interaction introduces a higher loading of Pd in Fe_3_O_4_@PUN-Pd(2) (3.05 wt%) compared to Fe_3_O_4_@PUN-Pd(0) (0.92 wt%), as observed by ICP-OES measurement. Therefore, the smaller size and higher loading amounts of Pd in Fe_3_O_4_@PUN-Pd(2) enabled the higher efficiency for 4-NP reduction. Also, the N atom in the triazine unit can possibly act as an active site for reduction to enhance the reduction rate. Furthermore, we also investigated the catalytic properties of Fe_3_O_4_@PUN-Pd(2) in the reduction of other nitroarene derivatives, such as 4-NA, 2-NA, NB and 2-NT, in aqueous medium. The reaction conditions, including the amount of catalyst and concentration, were kept constant. The reaction progress of these nitro-derivatives was monitored using UV-vis spectroscopy. The UV-visible spectra of various graphs are provided in Fig. S10, SI. In the case of 4-NA, 2-NA, NB and 2-NT, there is a conversion of 89%, 82%, 79% and 75%, respectively. We have also tried 4-NP reduction in other organic solvents such as ethanol, methanol and acetonitrile, which shows uneasy reduction with a lower conversion percentage (Fig. S11, SI). Based on the findings, it can be inferred that water serves as the most effective medium for the reduction process, which depicts the conversion of 97% for 4-NP in 7 min at RT. The polarity of water is higher than that of other organic solvents, which stabilises the charged intermediate species and enhances the rate of reaction. Furthermore, the lower solubility of NaBH_4_ in certain organic solvents compared to water inhibits 4-NP reduction. Moreover, the kinetic experiments demonstrate higher conversion rates in water, where the aqueous medium not only facilitates efficient catalytic activity but also improves reaction kinetics, resulting in fast reductions. Together, these aspects highlight the importance of utilising aqueous media from an efficiency perspective as well as from environmental and practical perspectives.

### 3.4 Catalytic mechanism and kinetic study

**Scheme 2:**
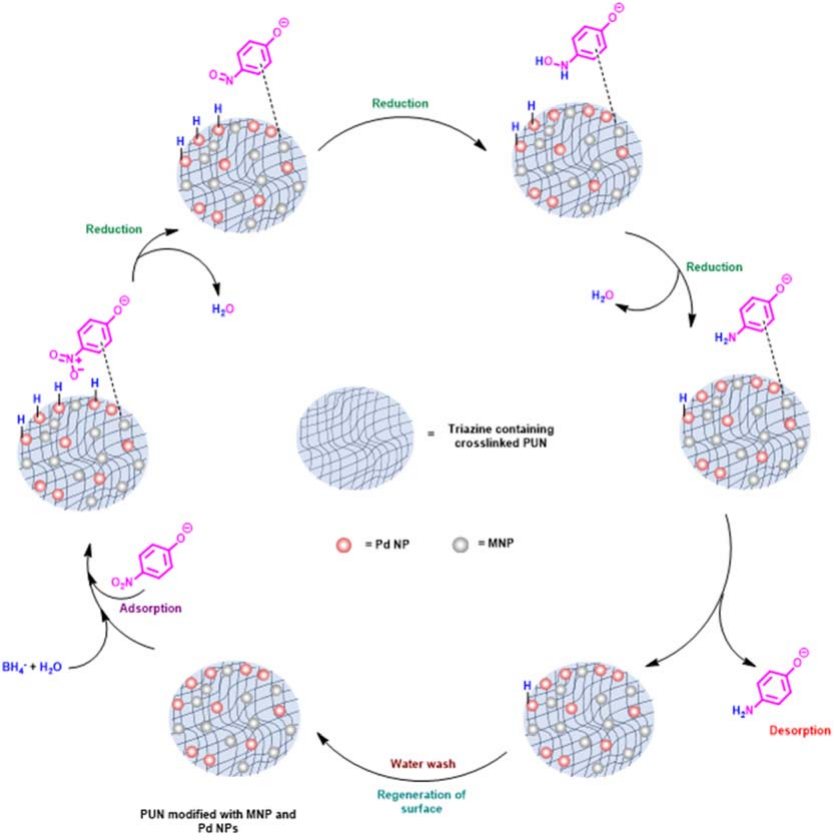
The plausible reaction mechanism of the reduction of 4-NP using PUN-modified MNP and Pd NP catalysts.

Polymer-immobilised metal catalysts offer an appropriate surface for reactants (*e.g.*, 4-NP and NaBH_4_), facilitating electron transfers that control reduction processes. The possible Langmuir–Hinshelwood (LH) mechanism is proposed in Scheme 2.^72^

Numerous reports on the same probable pathway over other catalysts support our hypotheses.^73,74^ Briefly, 4-nitrophenolate ions possibly gets adsorbed on the prepared catalyst. Simultaneously, Pd NPs react with NaBH_4_ to generate the Pd–H complex. Since a significant portion of the activated hydride on the catalyst's surface is lost as gaseous H_2_, this catalytic reaction often takes place with an excess of the hydride source NaBH_4_.^74^ The plausible reaction mechanism is depicted in Scheme 2, where the hydrides on the catalyst's surface are activated and transferred to the nitro group to produce the 4-aminophenolate ion. Finally, the 4-aminophenolate ion is desorbed from the catalyst. The same catalyst is recovered and reused for the next cycle after removing the leftover hydride adsorbed on the surface by water washing. Crucially, uniform distributions of Pd NPs on the catalyst surface strongly encourage the formation of Pd–H complexes.^75^ However, as we can see from the lack of change in the absorption peak, the reaction does not continue in the absence of a catalyst (Fig. 8a). In the presence of a catalyst and an excess of NaBH_4_, 4-NP is reduced by six electrons with the consumption of six protons (H^+^) (eqn (2)). Moreover, our catalyst contains acidic (Pd^2+^, Fe^2+^ and Fe^3+^) and basic (O^2−^) sites; while basic sites prefer to draw the dissociated hydrogen of NaBH_4_, acidic sites encourage the breakdown of the N–O bond in the intermediate phenylhydroxylamine.^76^ A six-electron process can be used to describe the entire hydrogenation.

2 C_6_H_5_NO_3_ + 6H^+^ + 6e^−^ → C_6_H_7_NO + 2H_2_O

**Fig. 9:**
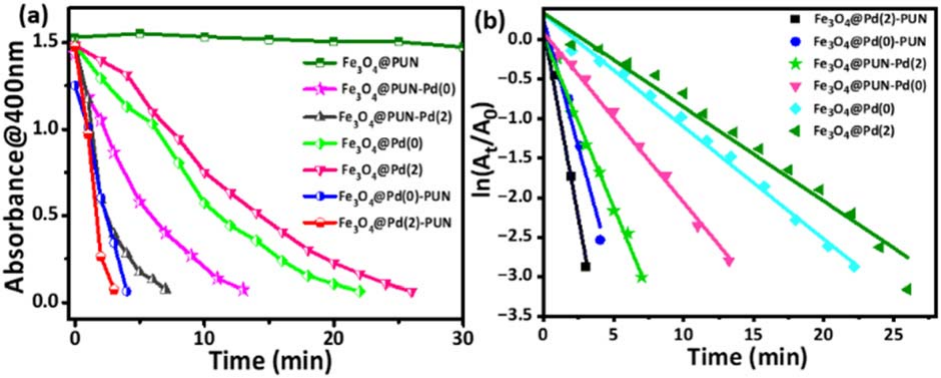
(a) Catalytic activity of different catalysts. (b) Linear plots of ln (*A*_t_/*A*_0_) *vs.* time for reduction of 4-NP by Fe_3_O_4_@PUN-Pd(0), Fe_3_O_4_@PUN-Pd(2), Fe_3_O_4_@Pd(0), Fe_3_O_4_@Pd(2), Fe_3_O_4_@Pd(0)-PUN, and Fe_3_O_4_@Pd(2)-PUN.

Significantly, the amount of reducing agent (NaBH_4_) is greater than the 4-NP concentration, which diminishes the influence of donor BH_4_^−^ on the catalytic activity. It means that the reaction is independent of the BH_4_^−^ concentration. Thus, these reactions follow a pseudo-first-order kinetic model (eqn (3)).^10,75^3
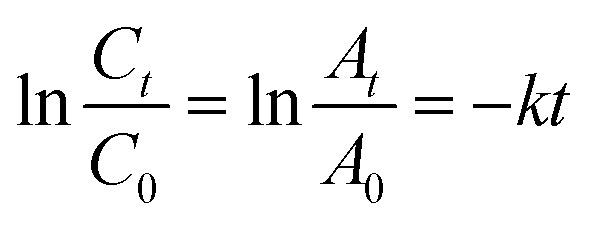
where *C*_t_ is the concentration at time interval *t*, *C*_0_ is the initial concentration, and *k* is the apparent rate constant. The catalytic performance is obtained from a linear relationship of ln (*A*_t_/*A*_0_) *versus* reaction time, shown in Fig. 9b, where *A*_t_ represents the absorbance at the time interval *t* and *A*_0_ is the absorbance at the initial stage of 4-nitrophenolate anions, respectively. The rate constants of Fe_3_O_4_@Pd(0)-PUN, Fe_3_O_4_@Pd(2)-PUN, Fe_3_O_4_@PUN-Pd(0), Fe_3_O_4_@PUN-Pd(2), Fe_3_O_4_@Pd(0) and Fe_3_O_4_@Pd(2) are 0.648, 0.965, 0.214, 0.430, 0.142 and 0.119 min^−1^, respectively, with a regression coefficient (*R*^2^) of more than 0.95 in all the cases, as shown in Table 1. Here, we observed that the reaction rate was affected by the presence of triazine-based PUN. In the absence of PUN, Fe_3_O_4_@Pd(0) and Fe_3_O_4_@Pd(2) showed lower rate constants compared to those catalysts with a triazine-containing unit (Fe_3_O_4_@PUN-Pd(0)/Pd(2), Fe_3_O_4_@Pd(0)/Pd(2)-PUN). The catalytic benchmarking of previously reported Fe_3_O_4_-supported Pd systems by Mahanitipong *et al.*,^77^ Fuchong Li and coworkers,^78^ and Yang *et al.*^79^ showed lower rate constants and TOF values when compared to our triazine-based polyurethane catalyst Fe_3_O_4_@PUN-Pd(2). Though the fundamental catalytic behaviour aligns with previously reported supported Fe_3_O_4_–Pd systems, our catalyst signifies the role of triazine, which may help in improving the intrinsic activity and stability of our Fe_3_O_4_@PUN-Pd(2) catalyst.

**Table 1:** Catalytic activity of different catalysts^a^

Type of catalyst	Reaction completion time (min)	Rate constant (*k*) (min^−1^)	Conversion (%)	Regression coefficient (*R*^2^)
Fe_3_O_4_@Pd(2)-PUN	4	0.965	95	0.988
Fe_3_O_4_@Pd(0)-PUN	3	0.648	94	0.962
Fe_3_O_4_@PUN-Pd(2)	7	0.430	97	0.993
Fe_3_O_4_@PUN-Pd(0)	13	0.214	97	0.995
Fe_3_O_4_@Pd(0)	22	0.142	97	0.960
Fe_3_O_4_@Pd(2)	26	0.119	97	0.966

a Reaction conditions: 40 mL of aqueous 4-NP (0.1 mM), 10 mL of freshly prepared NaBH_4_ (0.05 M) solution, and 10 mL of milliQ water at RT.

In addition, we scaled up the hydrogenation of various substituted nitroarenes, resulting in the formation of corresponding amines shown in Table S2, SI. The reaction was done by using 0.718 mmol of nitroarenes, 3.712 mmol of NaBH_4_ and 15 mg of catalyst at room temperature in water medium. Using thin-layer chromatography (TLC), the reaction progress was monitored. Once the reaction was complete, the product was extracted with ethyl acetate and dried over sodium sulfate. The formation of products was confirmed by different spectral analysis techniques such as ^1^H NMR, LC-MS and FT-IR (Fig. S12–S26, SI). All these results validate the formation of compounds without any impurities, which was observed by the formation of a single spot in TLC. The isolated yield was found to be in the range of 54–90% (Table S2, SI). The conversion and yield of various nitroaromatic compounds are listed in Table S3, SI. Nitroaromatic compounds with an electron-withdrawing group are susceptible to reduction. Among all nitroaromatic compounds mentioned in Table S3, SI, 4-NP exhibited a marginally electron-deficient nature due to the inductive effect of the electronegative O atom and also the presence of –OH, which made it more water-soluble. Therefore, 4-NP demonstrated the highest conversion (97%). Again, due to the presence of the hydrophobic –CH_3_ group, the water solubility of 2-NT is minimal, which is the reason for its lowest conversion (75%). The solubility of NB is low due to the absence of any polar substituent and hence the conversion value was also low (79%). Again, 4-NA and 2-NA have a similar structure. 2-NA demonstrates less conversion (82%) compared to 4-NA (89%) due to the steric effect near the –NO_2_ group. However, the isolated yield was shown to be less than the conversion (Table S3, SI). This could be because products were eliminated from the system with the aqueous layer during the workup process, while the reduced product was soluble in both water and organic media. Furthermore, during the catalyst separation process from the reaction medium, the generated product may be adsorbed on the catalyst's surface and eliminated from the reaction mixture.

Moreover, the turnover number (TON) of the catalyst Fe_3_O_4_@PUN-Pd(2) for 4-NP was calculated using eqn (4)4
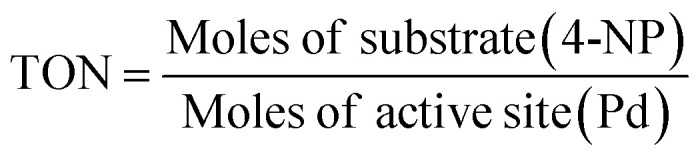


Similarly, the turnover frequency (TOF) value was also calculated using eqn (5).5
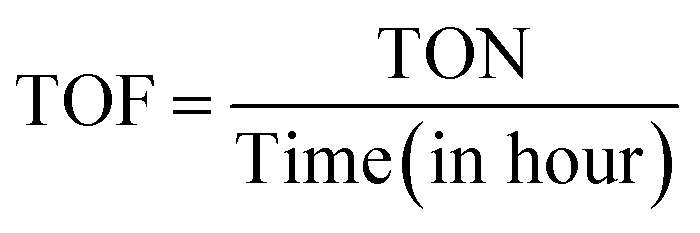


The catalyst Fe_3_O_4_@PUN-Pd(2) achieved a TON of 170 and a TOF of 257 h^−1^, respectively.

**Fig. 10:**
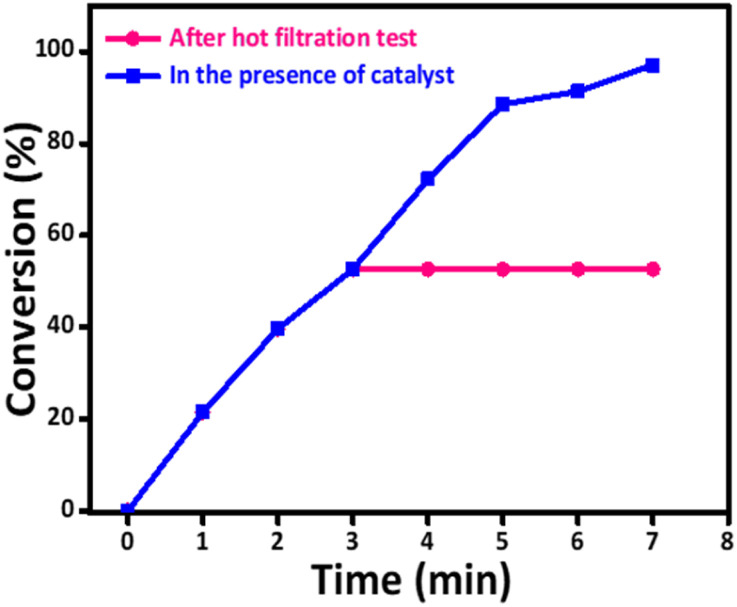
Hot filtration experiment to determine the heterogeneous nature of the Fe_3_O_4_@PUN-Pd(2) catalyst.

Furthermore, we have performed a detailed analysis of the prepared catalyst to determine its heterogeneous nature. In the first phase, a hot filtration study was conducted to assess the heterogeneity and stability of the catalyst (Fig. 10). The 4-NP reduction reaction was carried out by using a catalyst under standard optimised conditions. After 3 min of reaction, the catalyst was magnetically isolated from the reaction mixture. It was observed that the reduction was hampered at 52.58% conversion even after 7 min, whereas in the presence of catalyst, 97% conversion occurred. This indicates that there is no leaching of Pd, and the strong binding of Fe_3_O_4_, Pd NPs with PUN confirms the heterogeneity of the prepared catalyst. In the second phase, we have conducted a scavenger experiment to find the heterogeneous catalytic pathway of our system. The experiment was done by introducing scavengers like EDTA, PBQ and IPA to actively quench the reaction and checked its interference in the catalytic activity. As the prepared catalyst tended to be heterogeneous, the activity persisted without hindering the conversion of 4-NP in the presence of scavengers (Fig. S27a, SI). In the third case, the mercury poisoning mechanistic test was carried out to examine the heterogeneous pattern of the catalyst. Under model reaction conditions, the excess amount of Hg^2+^ (about 300 equivalents) was added to the reaction mixture after 4 min. In the presence of Hg^2+^, the 4-NP reduction rate drastically declined due to the formation of Pd–Hg amalgam with an increase in time. The reaction was stopped at 30 min as no further conversion from the poisoned catalyst was observed (Fig. S27b, SI). However, after the addition of Hg^2+^, a minor conversion was observed, which may be due to the deceleration of Pd–Hg amalgam formation during the reduction process when compared to the actual 4-NP reduction reaction. While the Hg poisoning experiment results are consistent with a predominantly heterogeneous catalytic route, they do not provide definitive proof for the complete absence of homogeneous Pd species.^80^ The Hg poisoning test has fundamental limitations, such as the possibility that mercury will deactivate extremely sensitive homogeneous clusters or conceal a tiny, parallel homogeneous cycle caused by trace leached palladium. Thus, in addition to the predominant heterogeneous mechanism, the presence of homogeneous Pd species is still possible. Nevertheless, the results obtained from heterogeneity experiments (hot filtration, scavenger and Hg poisoning) indicate that our prepared catalyst is predominantly heterogeneous in nature.

In order to assess the practical applications of the Fe_3_O_4_@PUN-Pd(2) catalyst for the removal of 4-NP from wastewater, specific 4-NP concentrations were added to tap, lake, and dam water, and the removal efficiency was examined with the same optimised parameters. The reduction of 4-NP using tap, dam and lake water is about 95.14%, 92.71% and 91.18%, respectively (Fig. S27c, SI).

### 3.5 Reusability studies

**Fig. 11:**
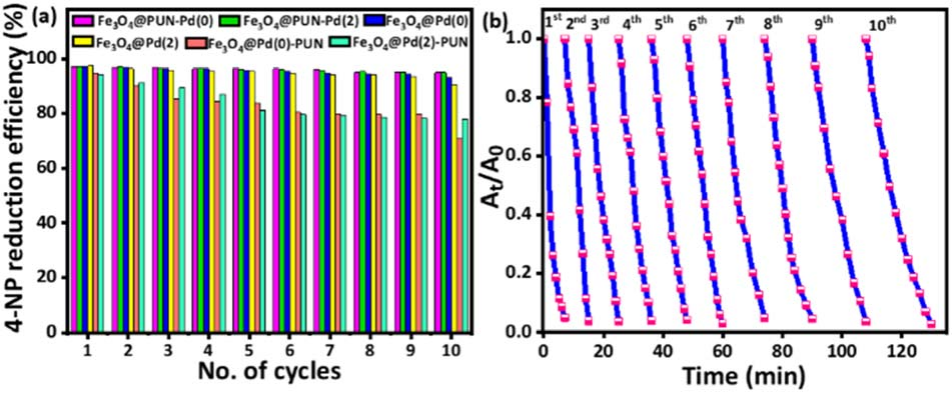
(a) Conversion of 4-NP with successive cycles of Fe_3_O_4_@PUN-Pd(0), Fe_3_O_4_@PUN-Pd(2), Fe_3_O_4_@Pd(0), Fe_3_O_4_@Pd(2), Fe_3_O_4_@Pd(0)-PUN, and Fe_3_O_4_@Pd(2)-PUN. (b) Plot of *A*_t_/*A*_0_*vs.* time up to 10 cycles for Fe_3_O_4_@PUN-Pd(2).

**Fig. 12:**
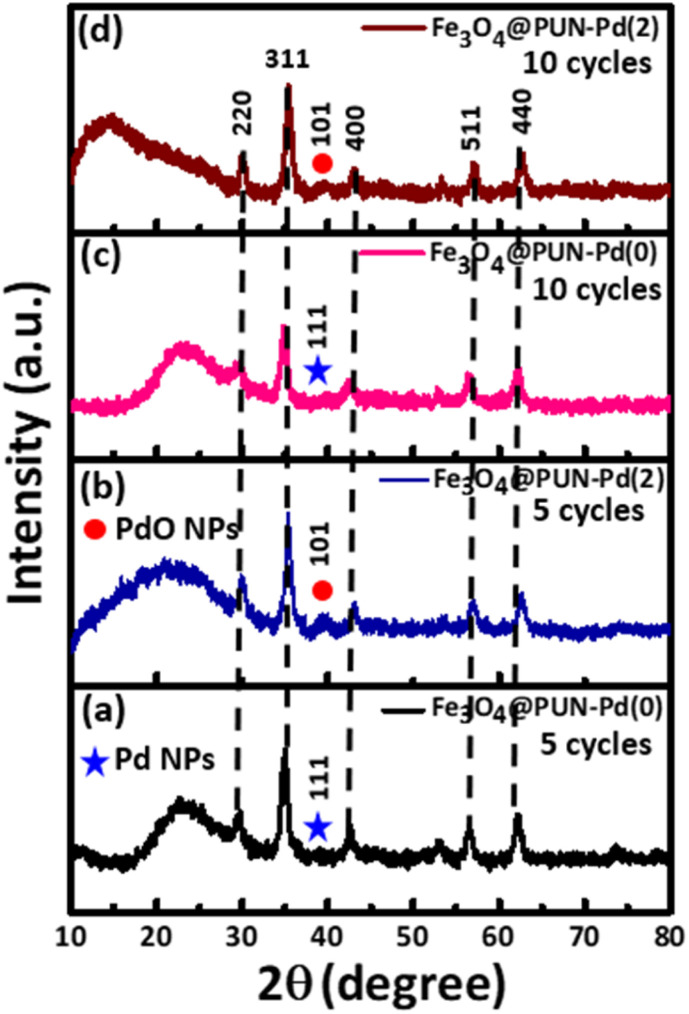
XRD patterns of (a) Fe_3_O_4_@PUN-Pd(0) and (b) Fe_3_O_4_@PUN-Pd(2) after five cycles. XRD patterns of (c) Fe_3_O_4_@PUN-Pd(0) and (d) Fe_3_O_4_@PUN-Pd(2) after 10 cycles.

One of the key factors to be examined under the same reaction conditions is the catalyst's stability and reusability. Fe_3_O_4_@PUN-Pd(0), Fe_3_O_4_@PUN-Pd(2), Fe_3_O_4_@Pd(0), Fe_3_O_4_@Pd(2), Fe_3_O_4_@Pd(0)-PUN and Fe_3_O_4_@Pd(2)-PUN were recycled using a magnet up to 10 cycles. Fe_3_O_4_@PUN-Pd(0) and Fe_3_O_4_@PUN-Pd(2) showed effective recyclability, and even after 10 cycles, the hydrogenation reaction exhibits no significant loss in catalytic activity with a good catalytic conversion of 95%. Whereas Fe_3_O_4_@Pd(0) and Fe_3_O_4_@Pd(2) exhibited catalytic conversion of 93% and 91%, respectively, after 10 cycles. Fe_3_O_4_@Pd(0)-PUN and Fe_3_O_4_@Pd(2)-PUN demonstrated 70% and 78% conversion, respectively, after 10 cycles (Fig. 11a). From these data, we conclude that the catalysts Fe_3_O_4_@PUN-Pd(0) and Fe_3_O_4_@PUN-Pd(2) are stable with better catalytic activity compared to other catalysts of our system. Yang *et al.* reported that the reduction was completed in one minute, but the catalyst can be reused only for five cycles, and it was recovered through centrifugation.^36^ Later, there is a decline in the activity of the catalyst due to possible complexation between 4-AP and Pd on the surface of the catalyst in their work. However, in our investigation, we can easily recycle the catalyst using a magnet, achieving 95% conversion even after 10 cycles. The catalytic activity of Fe_3_O_4_@Pd(0)-PUN and Fe_3_O_4_@Pd(2)-PUN was faster in the first cycle. However, during the recycling process, there might be a loss of catalyst, resulting in a decrease in the stability and activity of the catalyst. The linear plot of *A*_t_/*A*_0_*vs.* time up to 10 cycles for Fe_3_O_4_@PUN-Pd(2) is shown in Fig. 11b, and for other catalysts, the same is displayed in Fig. S28, SI. The enhanced catalytic activity and reusability of Fe_3_O_4_@PUN-Pd(2) compared to all other prepared catalysts are due to the synergistic effect of MNPs, PUN and Pd NPs. Furthermore, PUN might have stabilised MNPs electronically, which inhibits Pd NPs from clustering and leaching. The catalyst was able to be recycled and reused easily because of its magnetic feature, which made it simple to separate using an external magnetic field. The catalytic reactions were favoured by the even distribution of MNPs on the outer layer of the catalyst. The rigid, insoluble and infusible polymeric nature containing a triazine moiety possibly makes it convenient for recycling the catalyst with consistent catalytic activity. To further validate the stability of Fe_3_O_4_@PUN-Pd(2), the recovered catalysts were characterised by XRD, FT-IR, SEM, ICP-OES and XPS. XRD analysis of Fe_3_O_4_@PUN-Pd(0) and Fe_3_O_4_@PUN-Pd(2) was plotted after five cycles (Fig. 12a and b) and ten cycles (Fig. 12c and d), respectively.

There is no noticeable shift in the XRD peaks after five and ten cycles when compared to the fresh catalyst. FT-IR results of the catalyst after five and ten cycles are almost similar to the functional group of the fresh catalyst, retaining surface functional groups, without much change in the peak positions (Fig. S29, SI). The SEM images shown in Fig. S30a and S30b, SI were taken after five and ten cycles, respectively, for the Fe_3_O_4_@PUN-Pd(2) catalyst. No alteration in the morphology of the microspheres was observed after five cycles, whereas after ten cycles the surface and shape of the microspheres changed due to multiple recycling hydrogenation reactions and surface modification. ICP-OES was analysed to find the leaching of Pd; to our delight, there is not much loss in the Pd content after five cycles, obtaining ∼2.13 wt%, but minimal loss was observed after 10 cycles, around ∼1.56 wt% Pd. The XPS spectra of Fe_3_O_4_@PUN-Pd(0) after five and ten cycles are shown in Fig. S31a–S31c, SI. The peaks at 335.1 eV and 340.4 eV belong to Pd 3d_5/2_ and Pd 3d_3/2_ of Pd(0), respectively (Fig. S31b and S31c, SI). The peaks at 336.5 eV and 342.1 eV are assigned to Pd(2) of Fe_3_O_4_@PUN-Pd(2) (Fig. S31e and S31f, SI). This demonstrates the catalyst's consistency without metal leaching. However, the satellite peak at 346 eV after recycling is generated from charge-transfer processes and the oxidation of Pd NPs due to NaBH_4_ addition during the reduction process.^81^ The possible synergistic interaction between MNPs, PUN, and Pd NPs results in consistent catalytic activity and recyclability. In addition, PUN may stabilise MNPs electronically, inhibiting Pd NPs from aggregating and leaching. The even distribution of Pd NPs on the surface promotes catalytic processes. Moreover, the magnetic nature enables intuitive separation with an external magnet, facilitating recovery and reuse. Thus, the recommended Fe_3_O_4_@PUN-Pd(2) microspheres offer an opportunity for developing a metal nano-catalyst that is substantially potent, stable, and recyclable.

### 3.6 Catalytic performance comparison of Fe_3_O_4_@PUN-Pd(2) with various catalysts

The catalytic activity of Fe_3_O_4_@PUN-Pd(2) for the reduction of 4-NP is also compared with other previously reported catalysts (Table S4, SI). To explain the effectiveness of our system, we included some recently published PUN-supported metal catalysts, Pd@PUN, M/CH-PUS, and Ag-PU-S/Alg, where the PUN structure does not contain the triazine unit (entries 1–3). Although the conversion of these catalysts is high (100, 100 and 89.27%, respectively), they suffer from low recyclability (4, 3 and 0 cycles) compared to Fe_3_O_4_@PUN-Pd(2) (10 cycles). The strong interaction between Pd(ii) and triazine and the rigid PUN structure might have enhanced the stability of the catalyst and its recyclability. Moreover, Fe_3_O_4_@PUN-Pd(2) exhibited 95% conversion after 10 cycles, which implies that Pd NPs did not agglomerate, possibly due to the well dispersion of Pd NPs onto the polymeric surface. This study demonstrates a clear advantage of using the triazine unit in PUN. Furthermore, some recent polymer-supported magnetic Pd catalysts (PDOPA/PAAm@mag-rGO, MNP@polymer-Pd, Fe_3_O_4_@ CMC/PDEAEMA-Pd(100) and MNP2-Pd-MC) are summarised in the table (entries 4–8), which showed higher TOF values (6738, 3912, 386 and 305.82 h^−1^, respectively) compared to Fe_3_O_4_@PUN-Pd(2) (257 h^−1^). Again, catalysts Pd NPs@CHI, Pd/CNT/Fe_3_O_4_/GO and Pd/3D-AC (entries 10–12) displayed lower TOF values (25.14, 8.22 × 10^−5^ and 100.8 h^−1^, respectively) compared to Fe_3_O_4_@PUN-Pd(2) (257 h^−1^). Therefore, our catalyst, Fe_3_O_4_@PUN-Pd(2) revealed modest activity in terms of TOF values, which could be due to the metal active centre embedded in the crosslinked polymeric matrix. However, the proposed catalyst may not show completely new catalytic behaviour, but in terms of recyclability, Fe_3_O_4_@PUN-Pd(2) exhibited reusable property towards the higher side (10 cycles). As a result, Fe_3_O_4_@PUN-Pd(2) fulfilled our objective, which was to prepare a catalyst with multiple recyclability. Fe_3_O_4_@PUN-Pd(2) demonstrated 10 times repeatability with 95% conversion in the last run, which was a rather useful outcome.

## 4 Conclusion

The current research aims to synthesize easily separable heterogeneous catalysts using an economical and environmentally friendly synthetic approach to promote the development of sustainable technologies. Additionally, a simple and eco-friendly method was adopted to prepare a Pd NP decorated polymer magnetic catalyst, designated as Fe_3_O_4_@PUN-Pd(2), serving as a heterogeneous catalyst. To the best of our knowledge, triazine is used for the first time in PUN to prepare the catalyst for nitroaromatic reduction. SEM analysis revealed the formation of microspheres modified by Pd NPs and MNPs with an average diameter of 3.76 µm, and the average particle size of MNPs with Pd(2) obtained from TEM results was around 7.77 ± 1.97 nm. Notably, Fe_3_O_4_@PUN-Pd(2) exhibited good thermal stability and high catalytic activity (∼97% conversion) in the reduction of 4-NP to 4-AP, using NaBH_4_ as the reductant in an aqueous medium. The presence of polymer containing a triazine unit aids in preventing catalyst leaching and deformation. Furthermore, the synthesized catalyst Fe_3_O_4_@PUN-Pd(2) can be easily recovered up to 10 cycles using an external magnet, with minimal loss of catalytic activity. In addition to 4-NP, Fe_3_O_4_@PUN-Pd(2) demonstrated relatively good catalytic activity towards other nitroarenes, *viz.*, 4-NA, 2-NA, 2-NT and NB in aqueous medium. These findings emphasize the potential of green synthesis approaches for developing efficient heterogeneous catalysts that are both cost-effective and environmentally sustainable. The demonstrated recyclability and competent performance of the catalyst make it well-suited for applications in both industrial and laboratory settings. Overall, the study highlights the catalyst's potential, which can be used for the treatment of industrial wastewater containing 4-NP and related toxic nitroarene compounds, contributing to cleaner technologies and enhanced environmental protection.

## Author contributions

The manuscript was written through the contributions of all authors. All authors have approved the final version of the manuscript.

## Conflicts of interest

There are no conflicts to declare.

## Supplementary Material

NA-OLF-D6NA00220J-s001

## Data Availability

The data used to support the findings of this study are included within the article. Supplementary information (SI): ^1^H-NMR, LC-MS, FTIR, HRTEM, EDX analysis, XPS, catalytic activity, recyclability and comparison of catalytic performance. See DOI: https://doi.org/10.1039/d6na00220j.
