# Supplementary material for: Fe3O4/Pd NPs immobilized on triazine-based polyurethane microspheres as a magnetically recoverable catalyst for the reduction of nitroarenes
Source: Nanoscale Adv. 2026 Jun 19;8(14):4124–38. doi: 10.1039/d6na00220j (PMC13281397; doi:10.1039/d6na00220j)
Supplement: NA-008-D6NA00220J-s001 [file NA-008-D6NA00220J-s001.pdf]

Supplementary Information (SI) for Nanoscale Advances.
This journal is © The Royal Society of Chemistry 2026

Fe_3O_4/Pd NPs Immobilized on Triazine-Based Polyurethane Microspheres as a Magnetically Recoverable Catalyst for the Reduction of Nitroarenes

Sindhu I Sanakal,[a] Anubhab Das,[a] Rahul Badri,[b] Pradip Kar,[c] Susanta Banarjee[b] and Samarendra Maji[a]*

[a]Department of Chemistry, SRM Institute of Science and Technology, Kattankulathur-603203, Tamil Nadu, India,

[b]Materials Science Centre, Indian Institute of Technology Kharagpur, Kharagpur-721302, India.

[c]Department of Chemistry, Birla Institute of Technology, Mesra, Ranchi, Jharkhand-835215, India.

Table of Contents

Items	Description	Page No.
**Fig. S1.**	^{1}H NMR spectrum of 3-THA.	S3
**Fig. S2.**	FTIR spectra of (a) PUN, (b) Fe_3O_4@PUN, (c) Fe_3O_4@PUN-Pd(0), (d) Fe_3O_4@PUN-Pd(2), (e) Fe_3O_4@Pd(0), (f) Fe_3O_4@Pd(2), (g) Fe_3O_4@Pd(0)-PUN, (h) Fe_3O_4@Pd(2)-PUN.	S4
**Fig. S3.**	EDX patterns of PUN microspheres.	S5
**Fig. S4.**	Energy-dispersive X-ray spectroscopy (EDX) mapping of (a)Fe_3O_4@PUN, (b) Fe_3O_4@PUN-Pd(0) and (c) Fe_3O_4@PUN-Pd(2) containing the elements C, O, N, Pd, Fe.	S5
**Fig. S5.**	TEM images of (a) Fe_3O_4@PUN-Pd(0), (c) Fe_3O_4@PUN-Pd(2) and SAED pattern of (b) Fe_3O_4@PUN-Pd(0), (d) Fe_3O_4@PUN-Pd(2).	S6
**Fig. S6.**	TEM images of (a), (b) and (c) Fe_3O_4@Pd(0)-PUN and (d), (e) and (f) Fe_3O_4@Pd(2)-PUN.	S6
**Fig. S7.**	EDX patterns of (a) Fe_3O_4@PUN-Pd(0), (b) Fe_3O_4@PUN-Pd(2), (c) Fe_3O_4@Pd(0)-PUN and (d) Fe_3O_4@Pd(2)-PUN.	S7
**Table S1.**	Particle size, zeta potential and PDI values of the synthesised microspheres calculated from DLS measurement.	S7
**Fig. S8.**	XPS spectra of (a) Fe2p, (b) O1s, (c) C 1s belongs to Fe_3O_4@PUN-Pd(0) and (d) Fe2p, (e) O1s, (f) C 1s belongs to Fe_3O_4@PUN-Pd(2) respectively.	S8
**Fig. S9.**	(a) 4-NP reduction using metallic compounds, (b) Effect of catalyst with different amounts of loading and (c) effect of change in $NaBH_4$ concentration on the reduction of 4-NP by Fe_3O_4@PUN-Pd(2).	S8
**Fig. S10.**	Catalytic reduction of (a) 4-NP, (b) 4-NA, (c) 2-NA, (d) NB, (e) 2-NT and (f) their conversion (%) by Fe_3O_4@PUN-Pd(2).	S9
**Fig. S11.**	Catalytic reduction of 4-NP in different solvents (a) ethanol (b) methanol and (c) acetonitrile (d) their conversion (%) by Fe_3O_4@PUN-Pd(2).	S10
**Table S2.**	Fe_3O_4@PUN-Pd(2) catalyst used for hydrogenation of nitroarene derivatives and the respective TLCs.	S11
**Table S3.**	Comparative study between conversion and yield of the reduction of nitroaromatic compounds by using Fe_3O_4@PUN-Pd(2) catalyst.	S12
**Fig. S12-Fig. S26**	^{1}H NMR, LC-MS and FT-IR of nitroarene derivatives.	S13-S17
**Fig. S27**	(a) Scavenger test using EDTA, PBQ and IPA, (b) Hg poisoning test for conversion of 4-NP, and (c) Conversion of 4-NP from real water sources (milli-Q, tap water, dam water, and lake water).	S18
**Fig. S28.**	(a) Plot of A_t/A_0 vs time upto 10 cycles of (a) Fe_3O_4@Pd(0), (b) Fe_3O_4@Pd(2), (c) Fe_3O_4@Pd(0)-PUN, (d) Fe_3O_4@Pd(2)-PUN and (e) Fe_3O_4@PUN-Pd(0).	S19
**Fig. S29.**	FTIR spectra of Fe_3O_4@PUN-Pd(2) after five and ten cycles.	S20
**Fig. S30.**	SEM images and EDX spectra of Fe_3O_4@PUN-Pd(2) after 5 cycles (a,c) and 10 cycles (b,d), respectively.	S20
**Fig. S31.**	(a) XPS survey spectra of Pd(0) (b) after 5 cycles and (c) after 10 cycles of Fe_3O_4@PUN-Pd(0), (d) XPS survey spectra of Pd(2) (e) after 5 cycles and (f) after 10 cycles of Fe_3O_4@PUN-Pd(2).	S21
**Table S4.**	Comparison of catalytic performance of Fe_3O_4@PUN-Pd(2) with other reported catalyst	S21
	References	S22

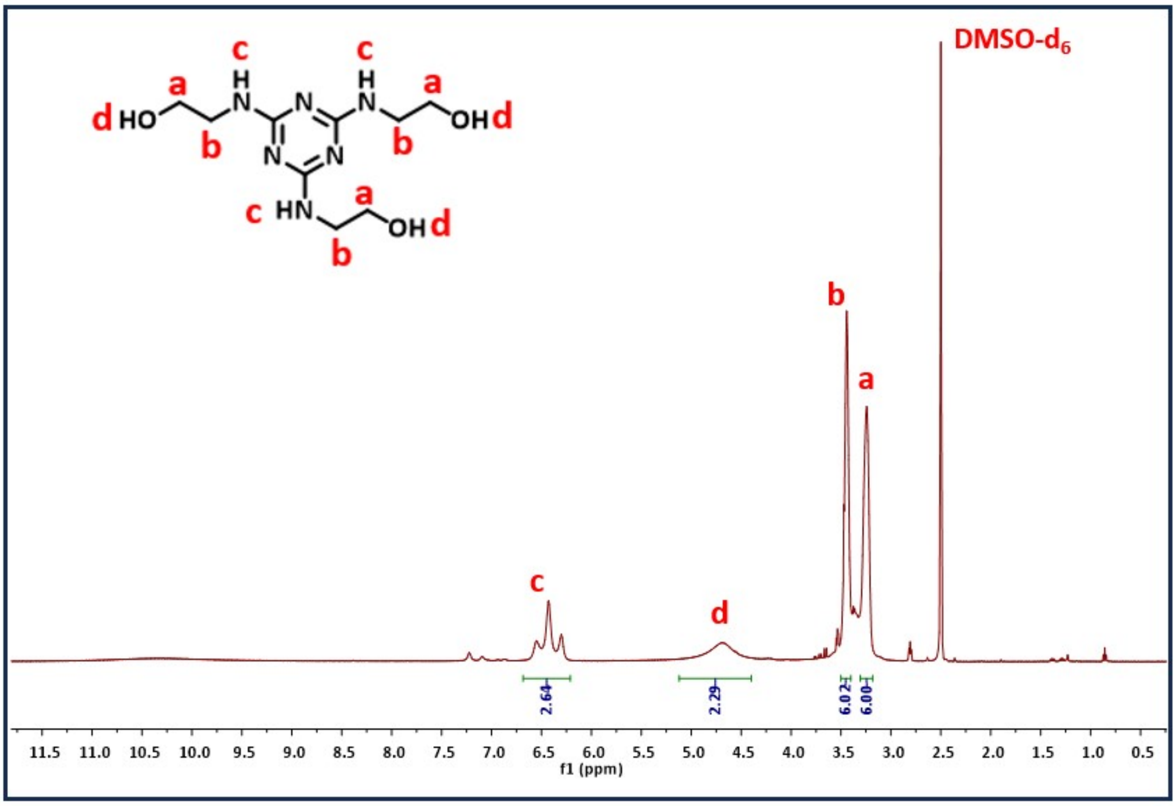

Fig. S1. ^{1}H NMR spectrum of 3-THA.

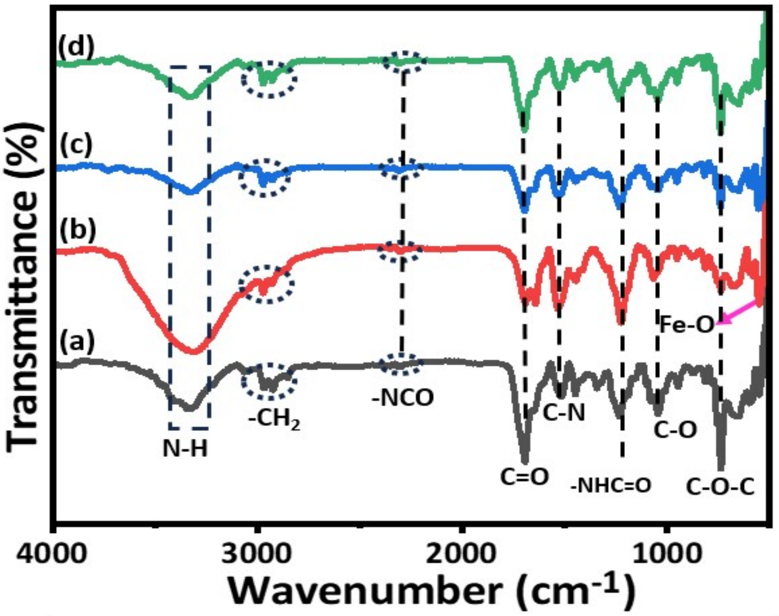

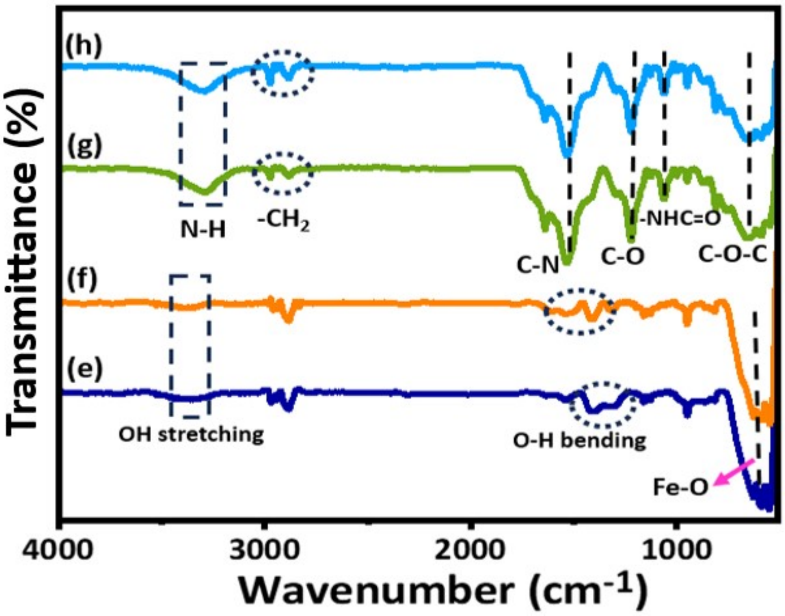

Fig. S2. FT-IR spectra of (a) PUN, (b) Fe_3O_4@PUN, (c) Fe_3O_4@PUN-Pd(0), (d) Fe_3O_4@PUN-Pd(2), (e) Fe_3O_4@Pd(0), (f) Fe_3O_4@Pd(2), (g) Fe_3O_4@Pd(0)-PUN, (h) Fe_3O_4@Pd(2)-PUN.

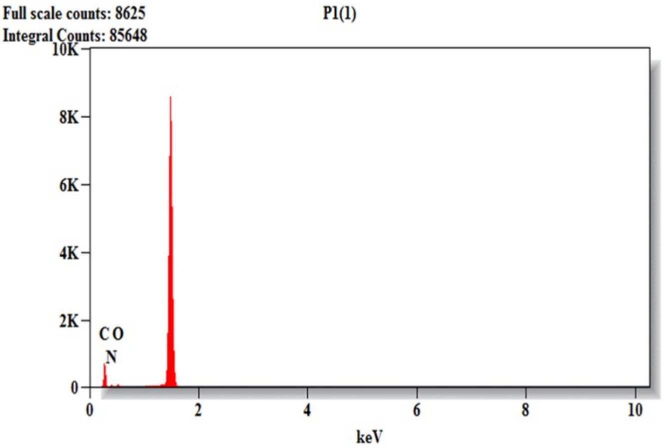

Element	Net Counts	Weight %	Atom %	Atom % Error	Formula
C	3624	36.21	41.45	± 0.67	C
N	402	30.59	30.03	± 3.73	N
O	552	33.20	28.53	± 2.58	O
Total		100.00	100.00		

Fig. S3. EDX pattern of PUN microspheres.

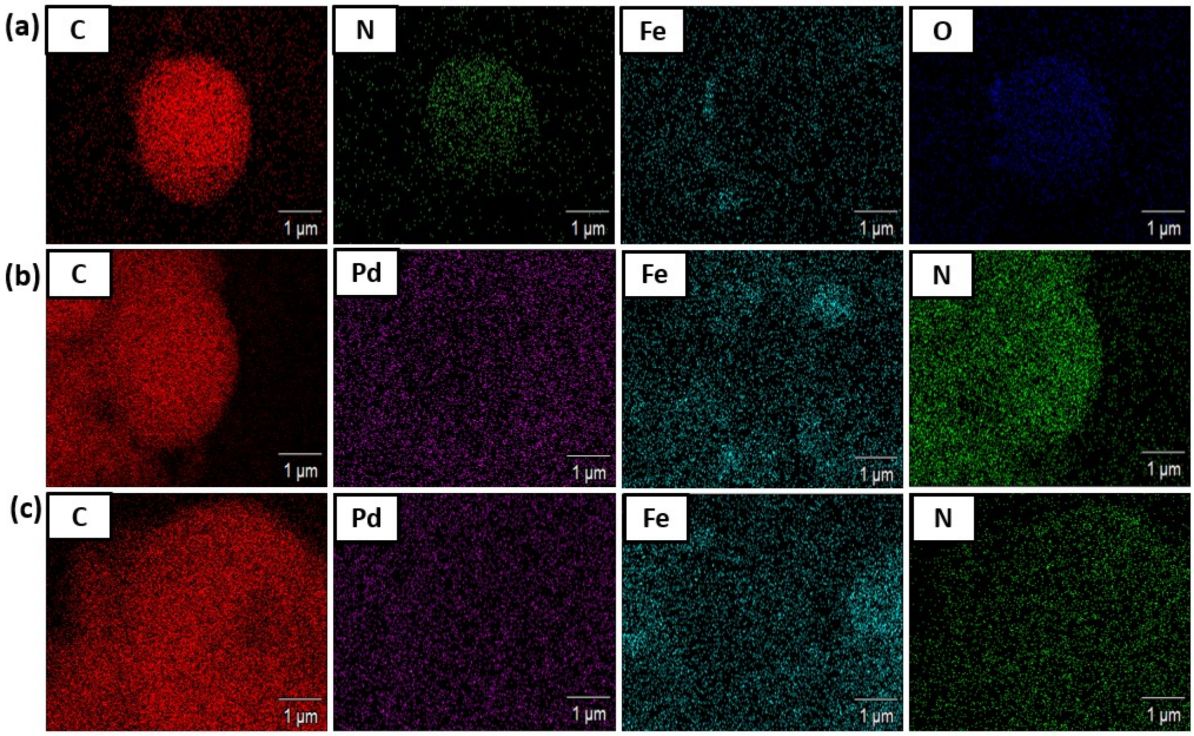

Fig. S4. Energy-dispersive X-ray spectroscopy (EDX) mapping of (a) Fe_3O_4@PUN, (b) Fe_3O_4@PUN-Pd(0) and (c) Fe_3O_4@PUN-Pd(2) containing the elements C, O, N, Pd, Fe.

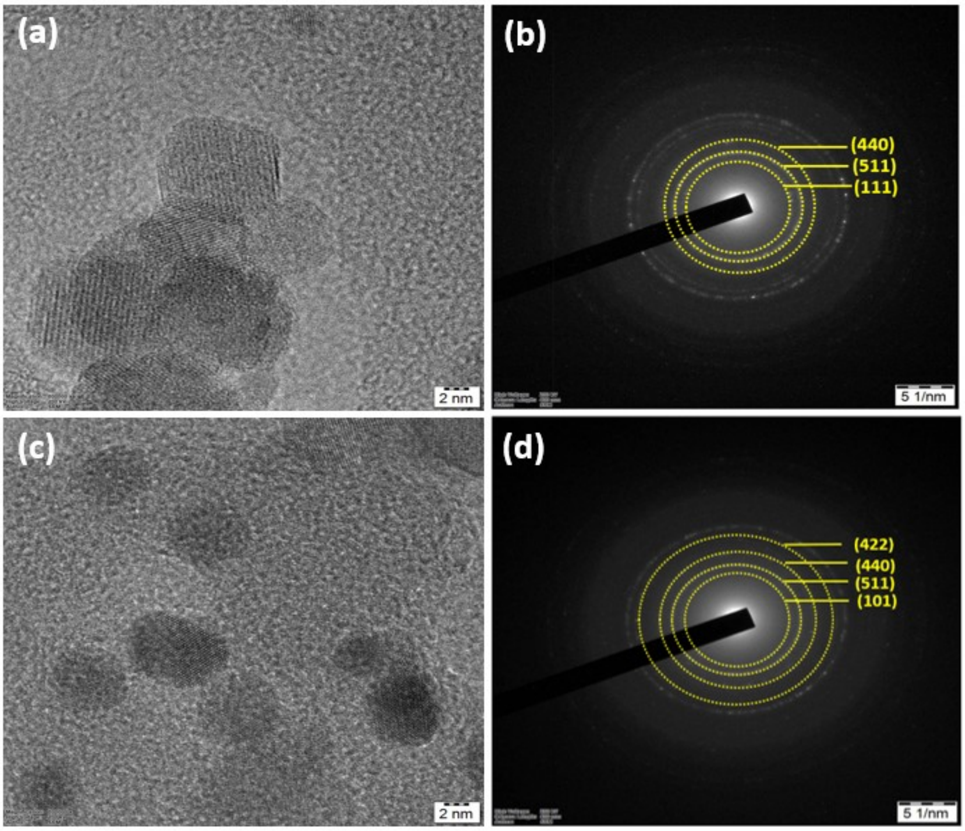

Fig. S5. TEM images of (a) Fe_3O_4@PUN-Pd(0), (c) Fe_3O_4@PUN-Pd(2) and SAED pattern of (b) Fe_3O_4@PUN-Pd(0), (d) Fe_3O_4@PUN-Pd(2).

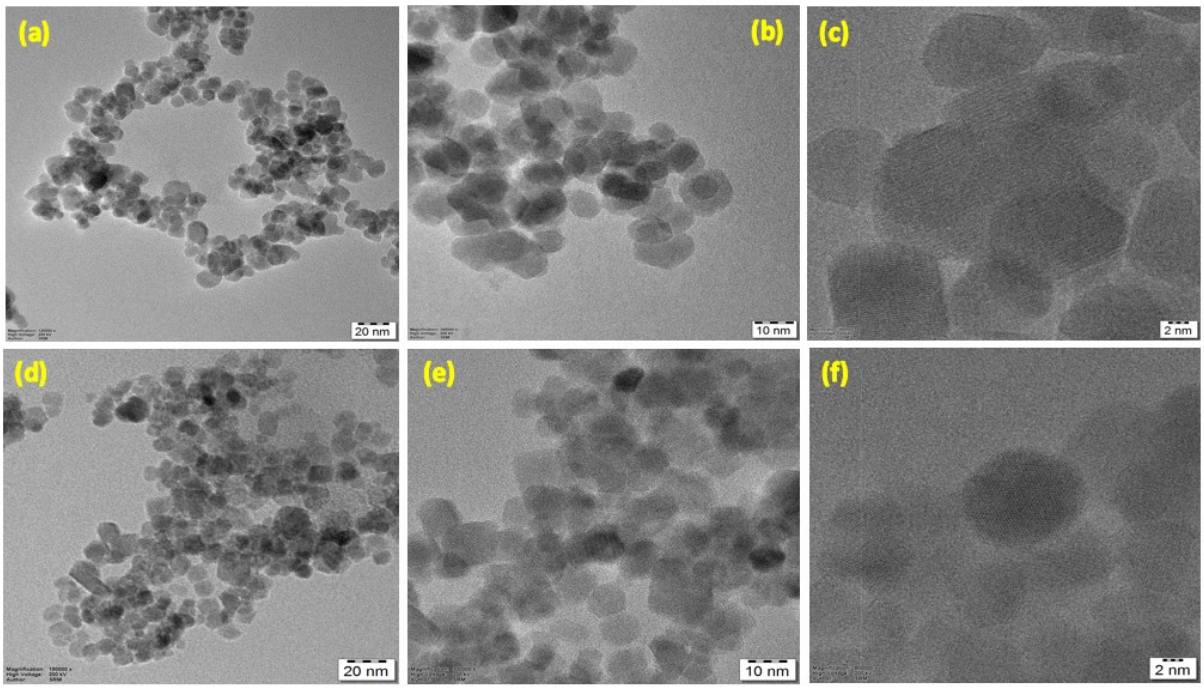

Fig. S6. TEM images of (a), (b) and (c) Fe_3O_4@Pd(0)-PUN and (d), (e) and (f) Fe_3O_4@Pd(2)-PUN.

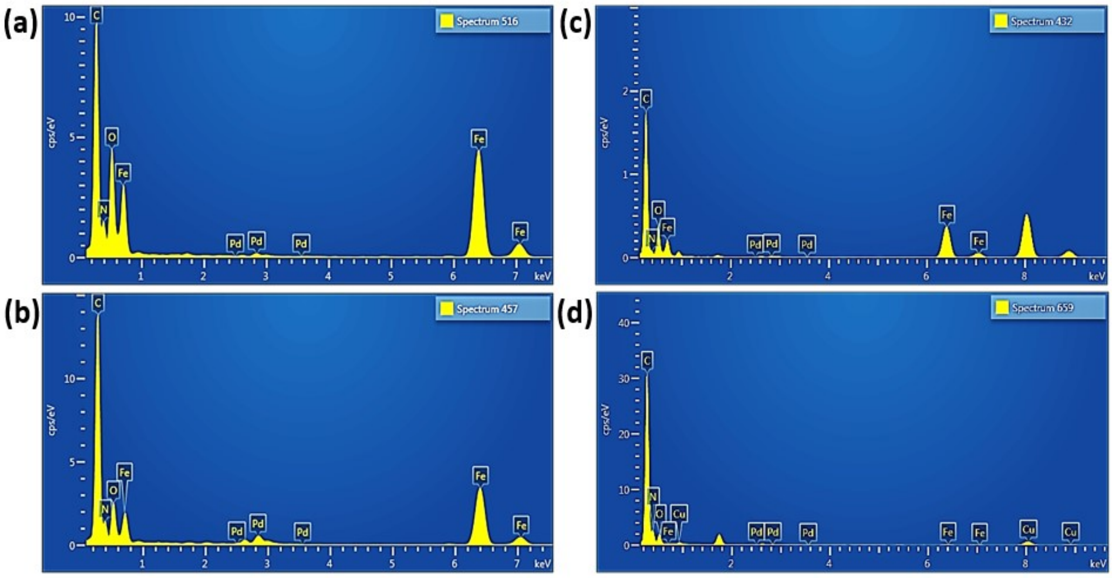

Fig. S7. EDX patterns of (a) Fe_3O_4@PUN-Pd(0), (b) Fe_3O_4@PUN-Pd(2), (c) Fe_3O_4@Pd(0)-PUN and (d) Fe_3O_4@Pd(2)-PUN.

Table S1. Particle size, zeta potential and PDI values of the synthesised microspheres calculated from DLS measurement.

Samples	Z-average particle size (nm)	PDI	Zeta potential (mV)
PUN	3023 ± 277.13	0.11	-5.70 ± 0.43
Fe_3O_4@PUN	3286 ± 509.30	0.13	-5.25 ± 0.10
Fe_3O_4@PUN-Pd(0)	3670 ± 718.99	0.42	-8.96 ± 0.51
Fe_3O_4@PUN-Pd(2)	1712 ± 170.42	0.21	6.76 ± 0.79
Fe_3O_4@Pd(0)	1461 ± 41.76	0.10	3.60 ± 0.42
Fe_3O_4@Pd(0)-PUN	1605 ± 359.36	0.47	-2.15 ± 0.55
Fe_3O_4@Pd(2)	520 ± 189.39	0.27	9.55 ± 0.60
Fe_3O_4@Pd(2)-PUN	1831 ± 163.33	0.29	5.88± 0.53

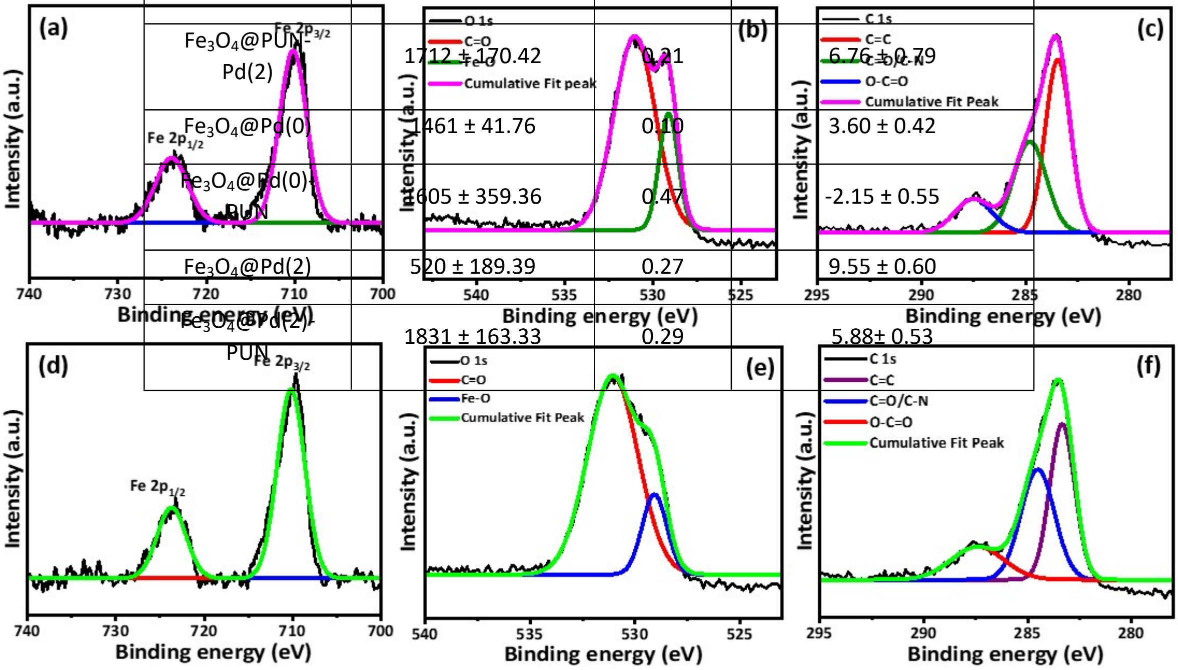

Fig. S8. XPS spectra of (a) Fe2p, (b) O1s, (c) C 1s belongs to Fe_3O_4@PUN-Pd(0) and (d) Fe2p, (e) O1s, (f) C 1s belongs to Fe_3O_4@PUN-Pd(2) respectively.

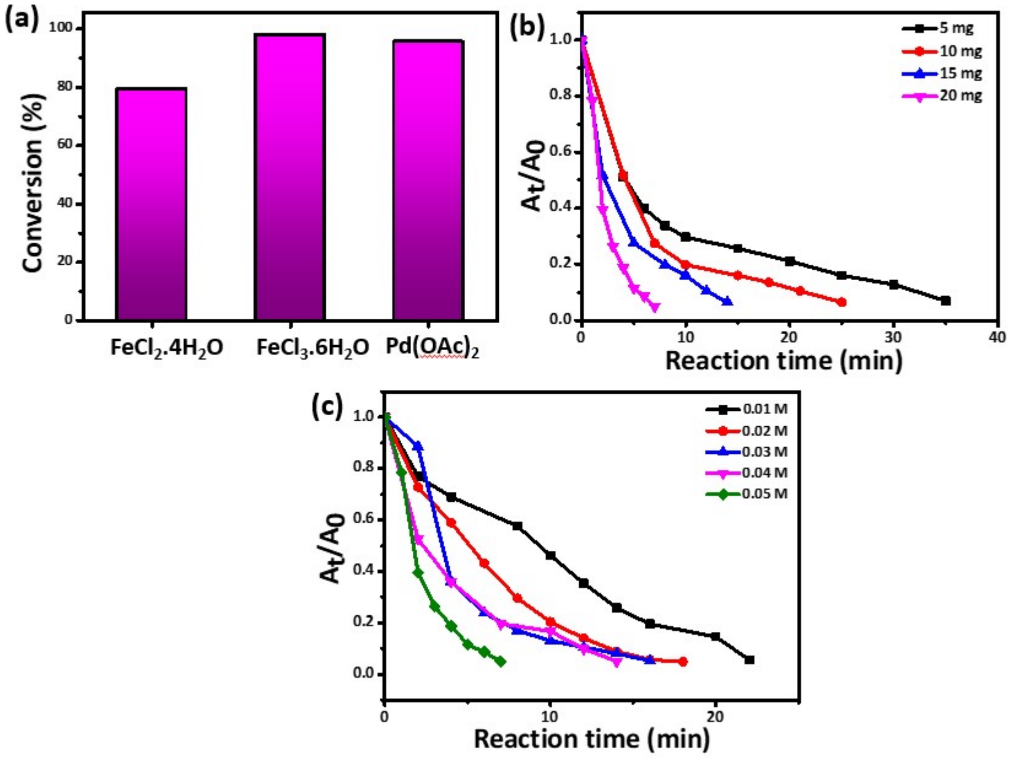

Fig. S9. (a) 4-NP reduction using metallic compounds, (b) Effect of catalyst with different amounts of loading and (c) effect of change in $NaBH_4$ concentration on the reduction of 4-NP by Fe_3O_4@PUN-Pd(2).

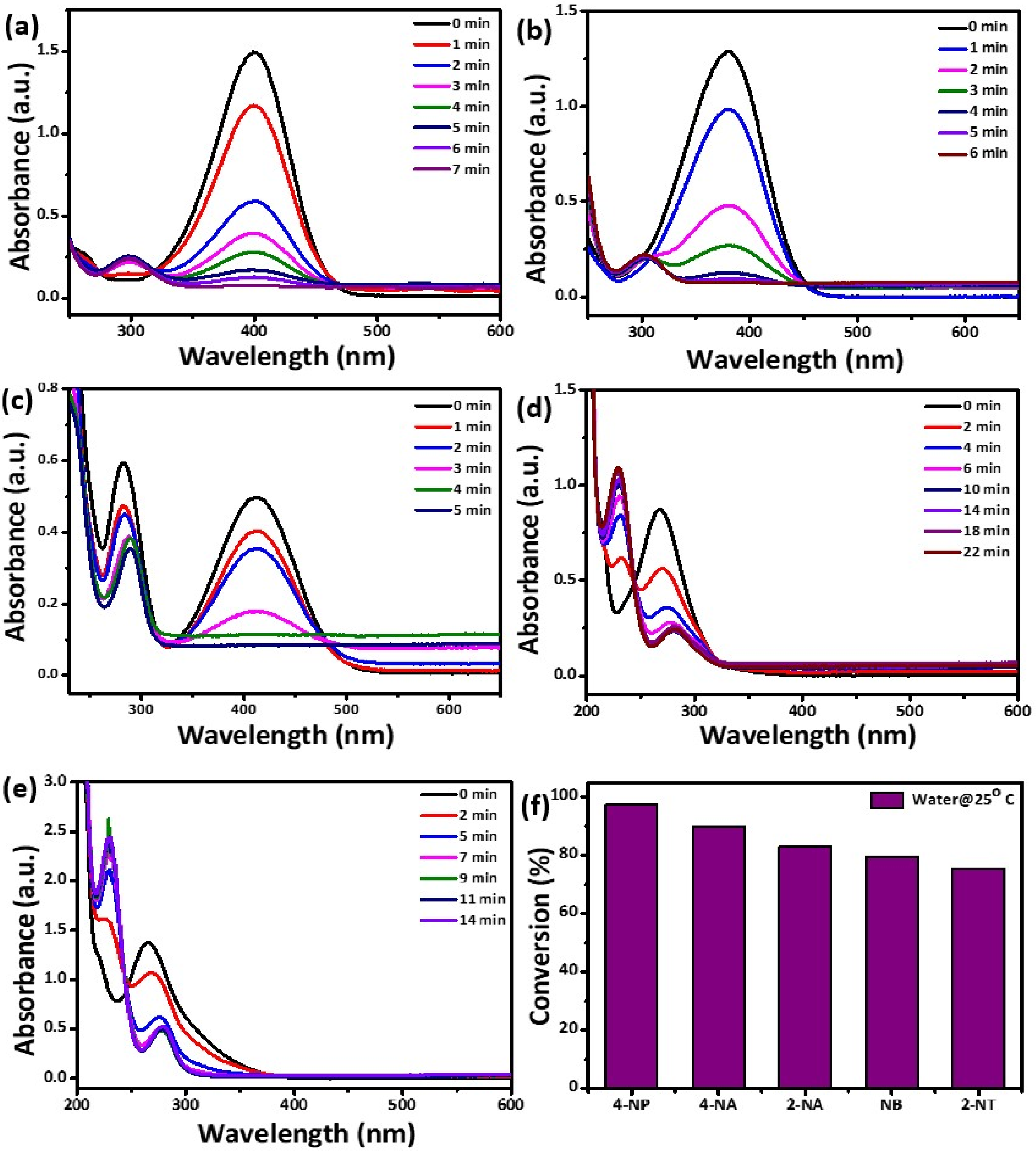

Fig. S10. Catalytic reduction of (a) 4-NP, (b) 4-NA, (c) 2-NA, (d) NB, (e) 2-NT and (f) their conversion (%) by Fe_3O_4@PUN-Pd(2).

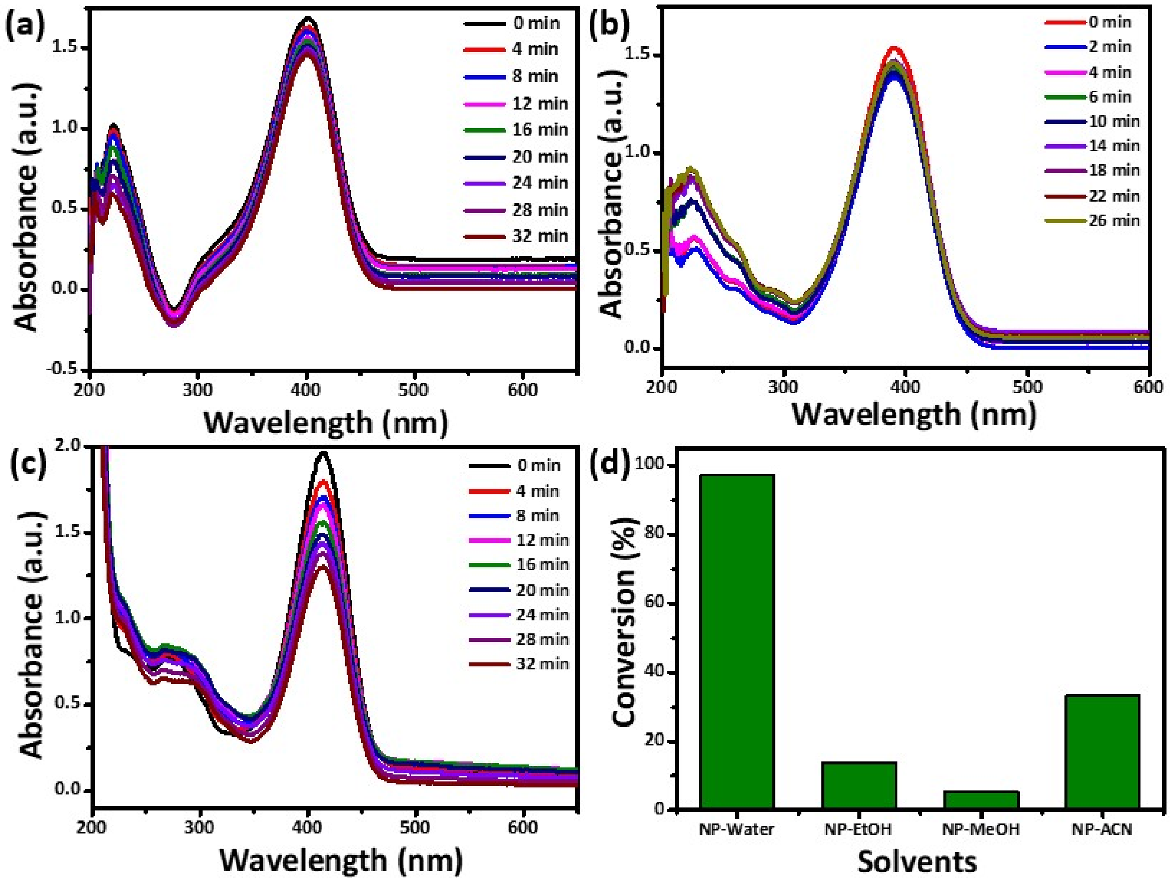

Fig. S11. Catalytic reduction of 4-NP in different solvents (a) ethanol (b) methanol and (c) acetonitrile and (d) their conversion (%) by Fe_3O_4@PUN-Pd(2).

Table S2 Fe_3O_4@PUN-Pd(2) catalyst used for hydrogenation of nitroarene derivatives and the respective TLCs (where S is the starting material and P/R is the product).

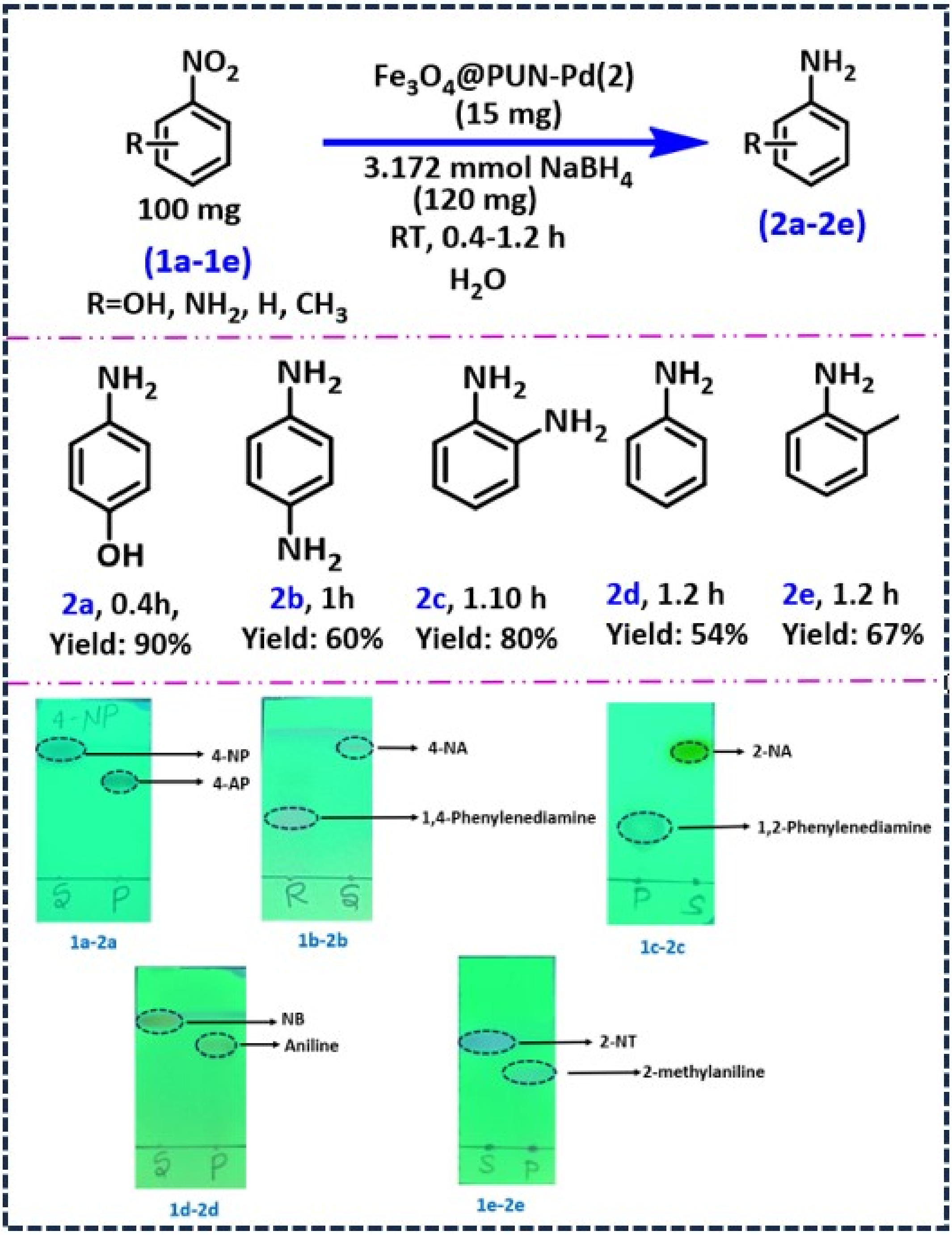

Table S3. Comparison study between conversion and yield of the reduction of nitroaromatic compounds by using Fe_3O_4@PUN-Pd(2) catalyst.

Entry	Nitroaromatic compound	Reduced product	Conversion (%)[a]	Yield (%)[b]
1	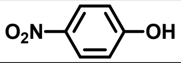	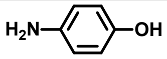	97	95
2	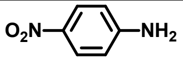	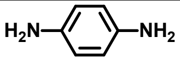	89	60
3	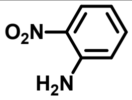	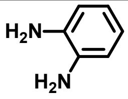	82	80
4	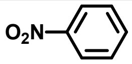	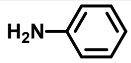	79	54
5	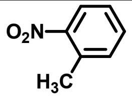	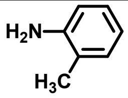	75	67

[a] Conversion was calculated from UV-*vis* spectroscopy. Reaction conditions: 40 mL of aqueous nitroaromatic compound (0.1 mM), 10 mL of freshly prepared $NaBH_4$ (0.05 M) solution, 10 mL of milliQ water at RT.

[b]Yield was calculated by performing the reaction at room temperature, as mentioned in Table S2.

Spectral analysis of nitroarene derivatives:

1. **4-Aminophenol (2a):** ^{1}H NMR (500 MHz, DMSO-d$_6$, δ): 4.34 (s, 2H), 6.37-6.39 (d, 2H), 6.43-6.45 (d, 2H); FT-IR: 3340, 3284, 1597, 1236, 1037 cm^{-1}.
2. **1,4-Phenylenediamine (2b)**: ^{1}H NMR (500 MHz, DMSO-d$_6$, δ): 4.15 (s, 2H), 6.32 (s, 4H); FT-IR: 3387, 3329, 1629, 1263 cm^{-1}.
3. **1,2-Phenylenediamine (2c)**: ^{1}H NMR (500 MHz, DMSO-d$_6$, δ): 4.38 (s,4H), 6.35-6.39 (m, 2H), 6.44-6.51 (m, 2H); FT-IR: 3381,1643, 1263 cm^{-1}.
4. **Aniline (2d)**: ^{1}H NMR (500 MHz, DMSO-d$_6$, δ): 4.95 (s, 2H), 6.40-6.58 (m, 3H), 6.90-7.04 (t, 2H); FT-IR: 3352, 3215, 1608, 1274 cm^{-1}.
5. **2-Methylaniline (2e)**: ^{1}H NMR (500 MHz, DMSO-d$_6$, δ): 2.10 (s, 3H), 4.75 (s, 2H), 6.34-6.48 (m, 1H), 6.53-6.58 (m, 1H), 6.82-6.90 (m, 2H). FT-IR: 3363, 3219, 2970, 1624, 1269 cm^{-1}.

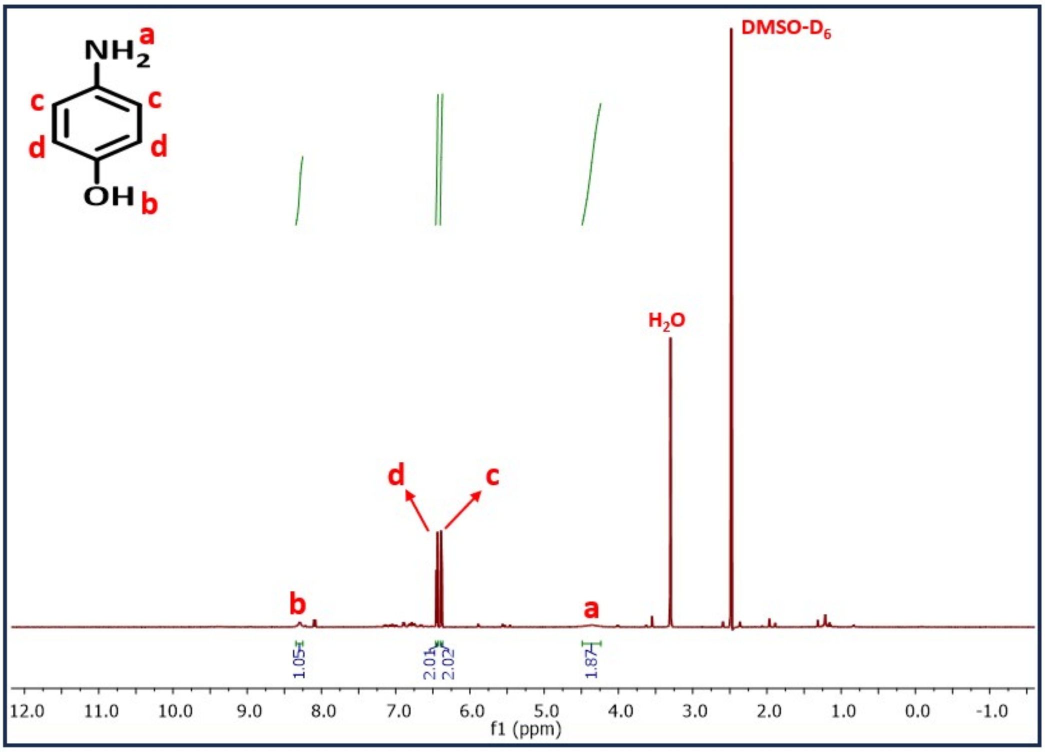

Fig. S12. ^{1}H NMR of 4-aminophenol.

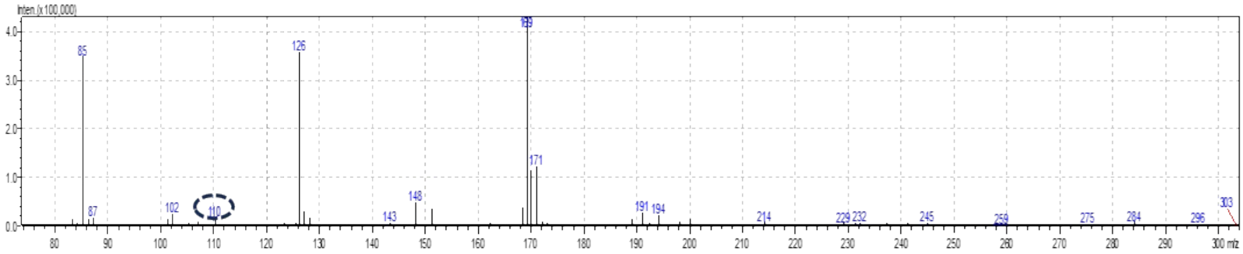

Fig. S13. HRMS spectrum of 4-aminophenol.

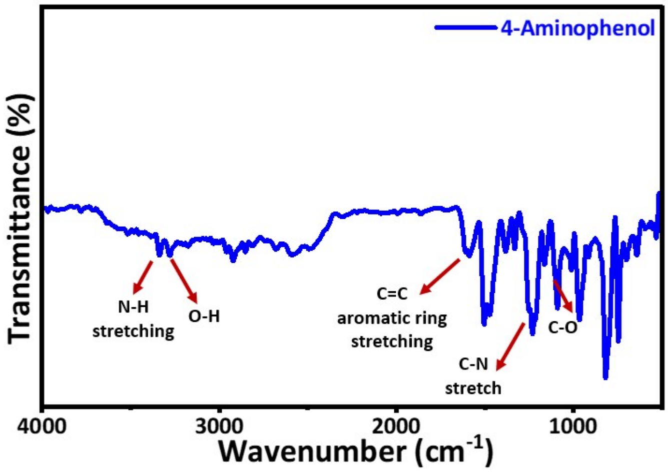

Fig. S14. FT-IR spectrum of 4-aminophenol.

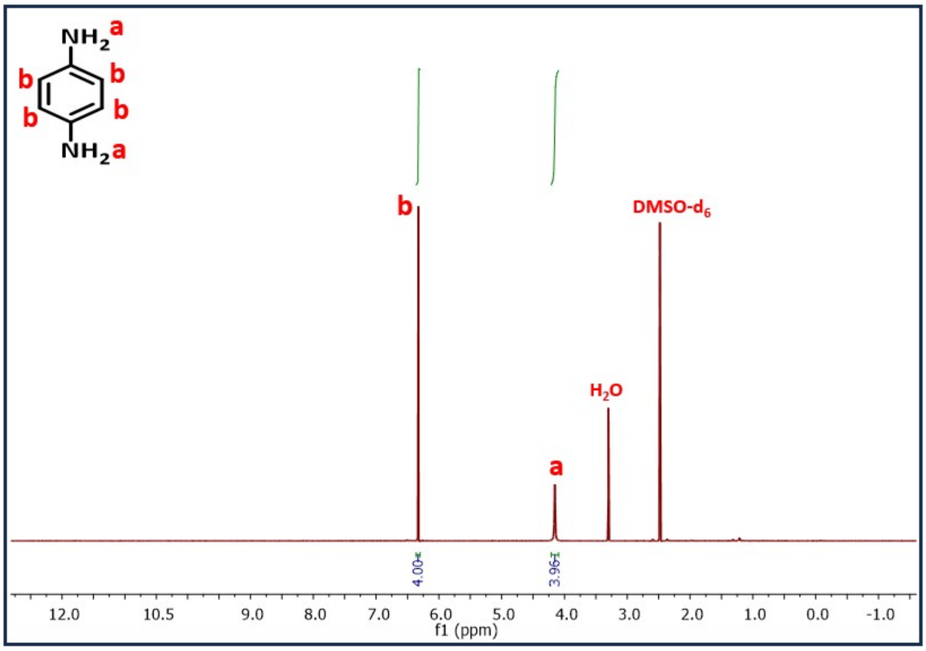

Fig. S15. ^{1}H NMR of 1,4-phenylenediamine.

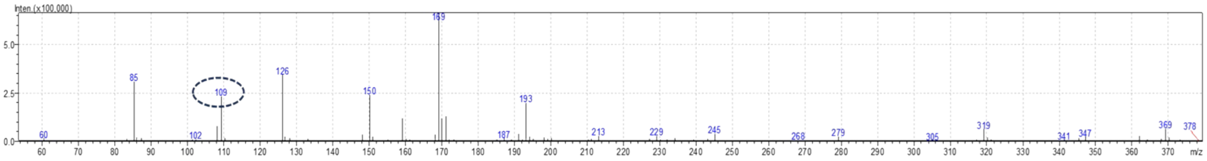

Fig. S16. HRMS spectrum of 1,4-phenylenediamine.

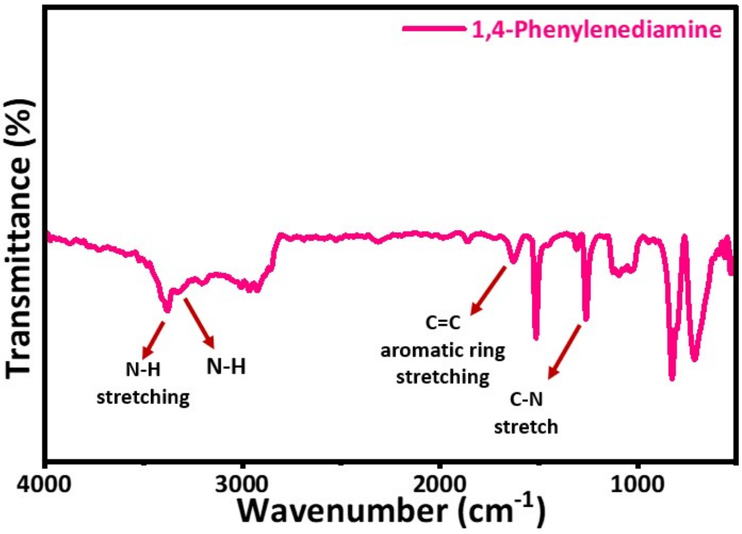

Fig. S17. FT-IR spectrum of 1,4-phenylenediamine.

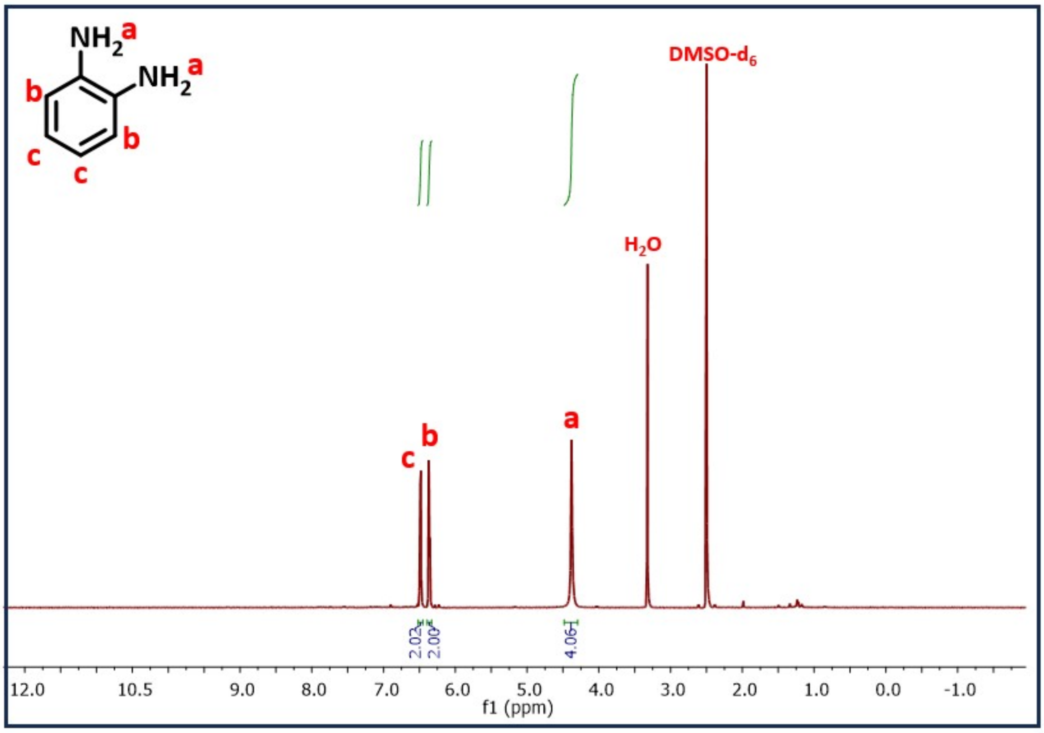

Fig. S18. ^{1}H NMR of 1,2-phenylenediamine.

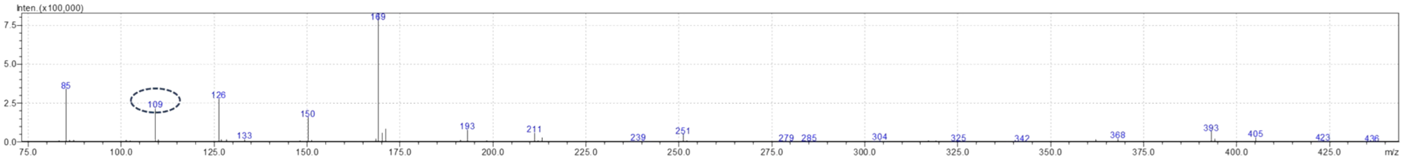

Fig. S19. HRMS spectrum of 1,2-phenylenediamine.

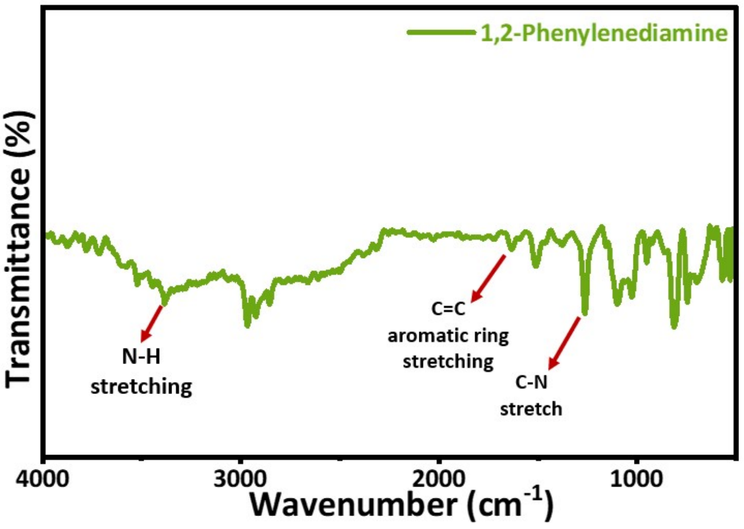

Fig. S20. FT-IR spectrum of 1,2-phenylenediamine.

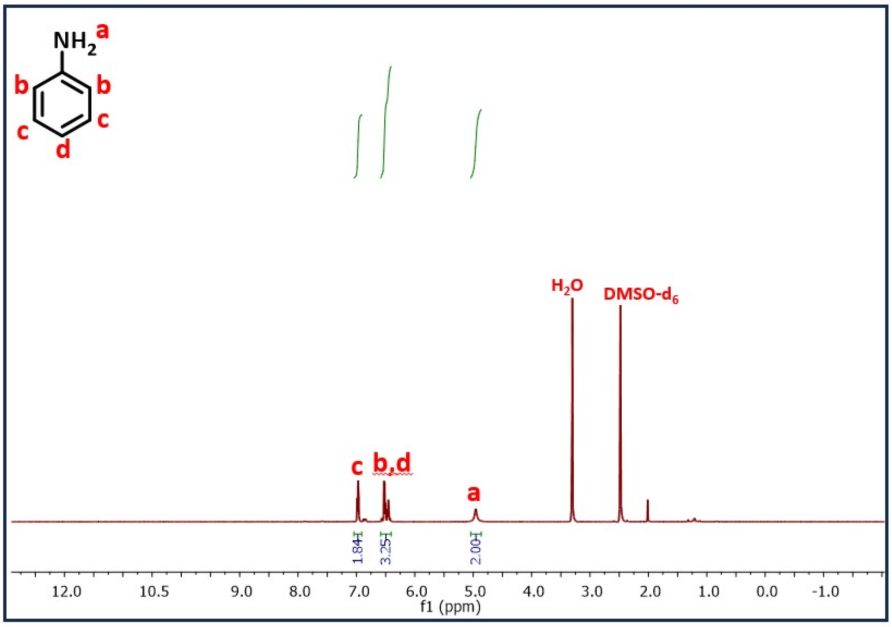

Fig. S21. ^{1}H NMR of aniline.

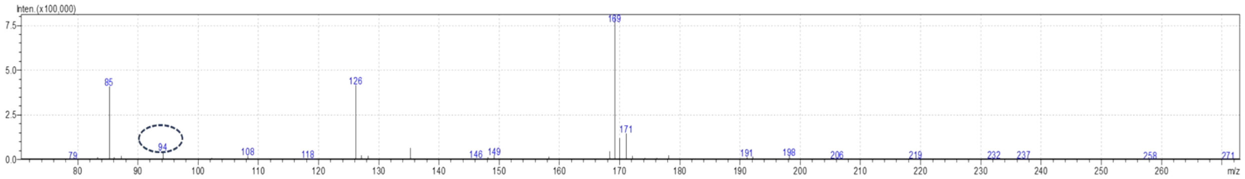

Fig. S22. HRMS spectrum of aniline.

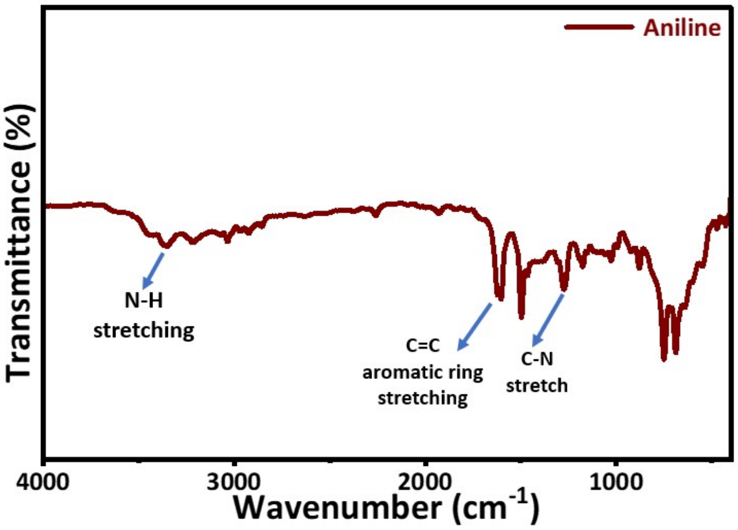

Fig. S23. FT-IR spectrum of aniline.

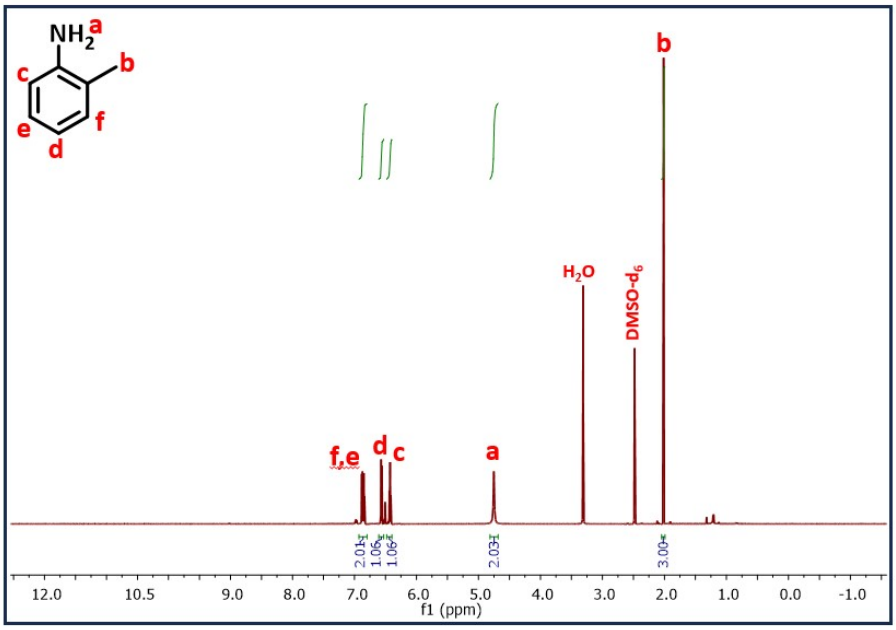

Fig. S24. ^{1}H NMR of 2-methylaniline.

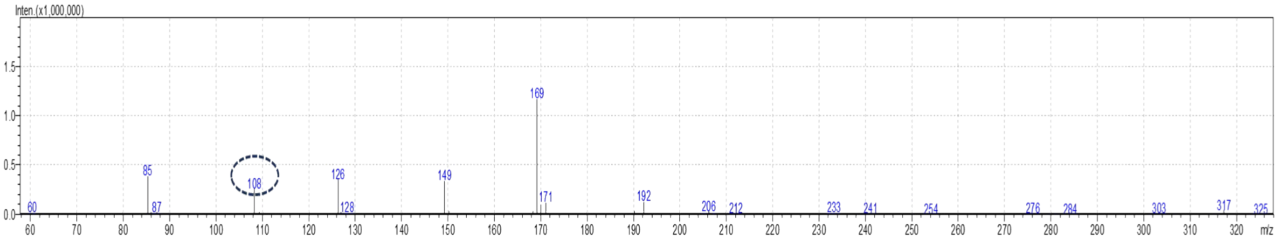

Fig. S25. HR-MS spectrum of 2-methylaniline.

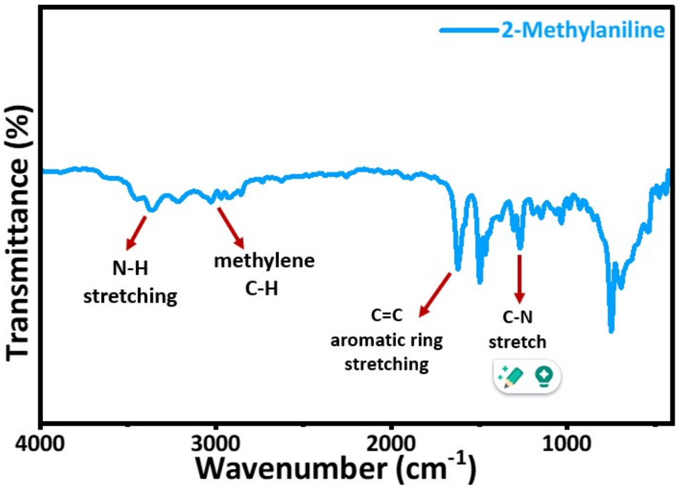

Fig. S26. FT-IR spectrum of 2-methylaniline.

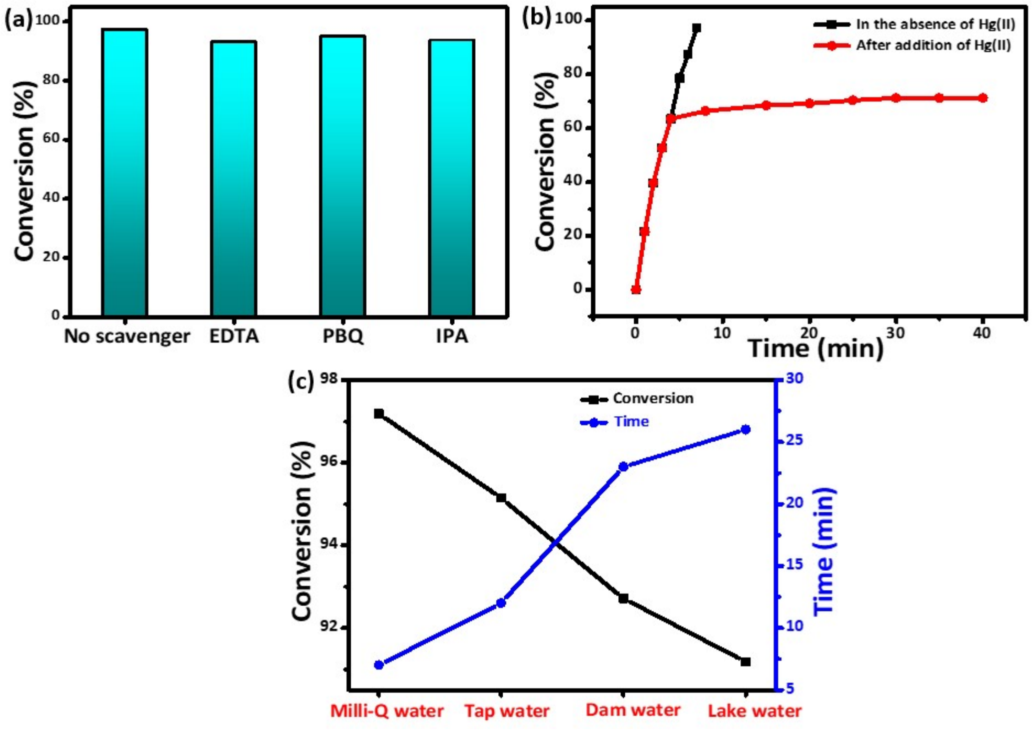

Fig. S27. (a) Scavenger test using EDTA, PBQ and IPA, (b) Hg poisoning test for Conversion of 4-NP, and (c) Conversion of 4-NP from real water sources (milli-Q, tap water, dam water, and lake water)

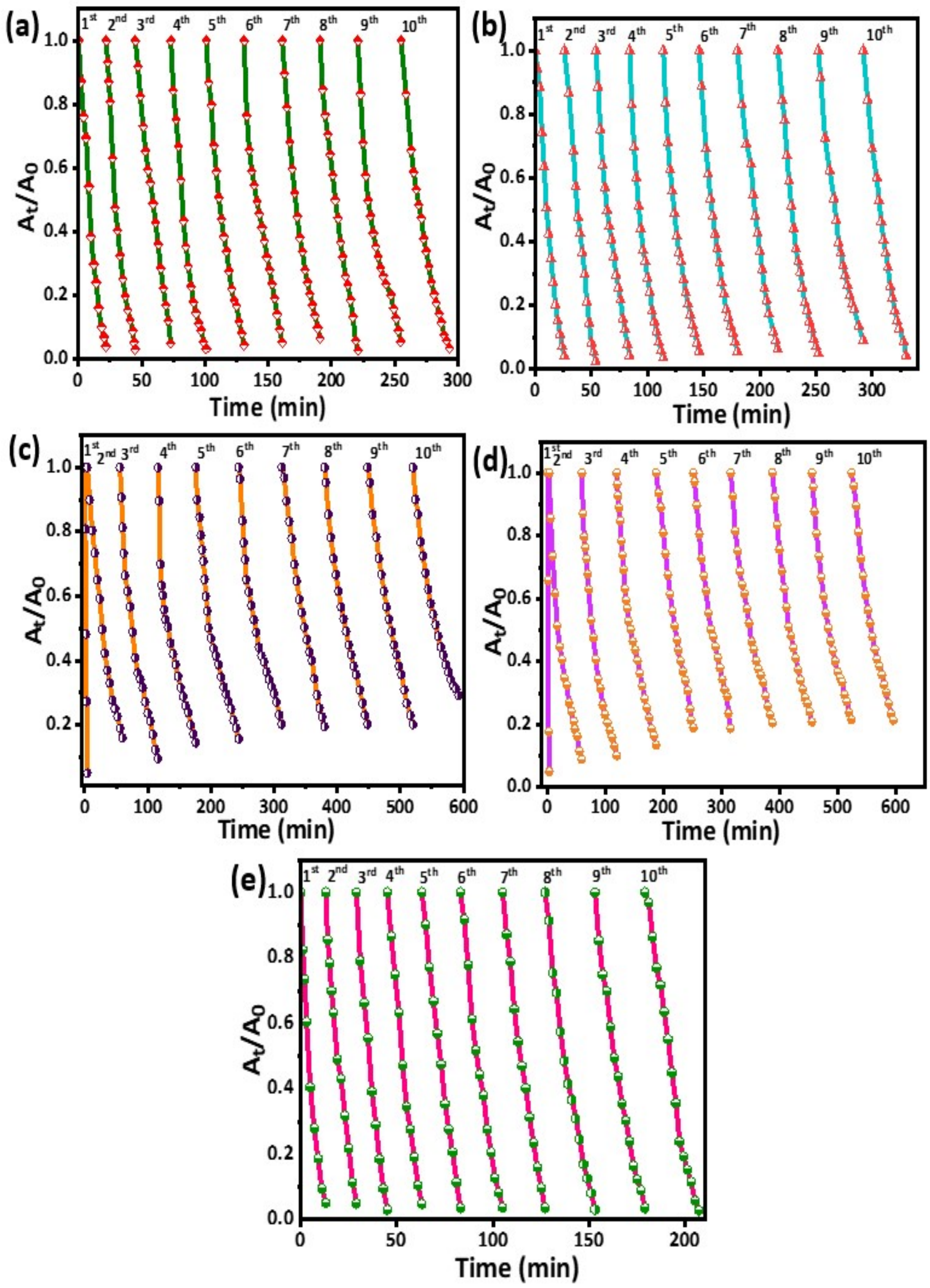

Fig. S28. Plot of A_t/A_0 *vs* time upto 10 cycles of (a) Fe_3O_4@Pd(0), (b) Fe_3O_4@Pd(2), (c) Fe_3O_4@Pd(0)-PUN, (d) Fe_3O_4@Pd(2)-PUN and (e) Fe_3O_4@PUN-Pd(0).

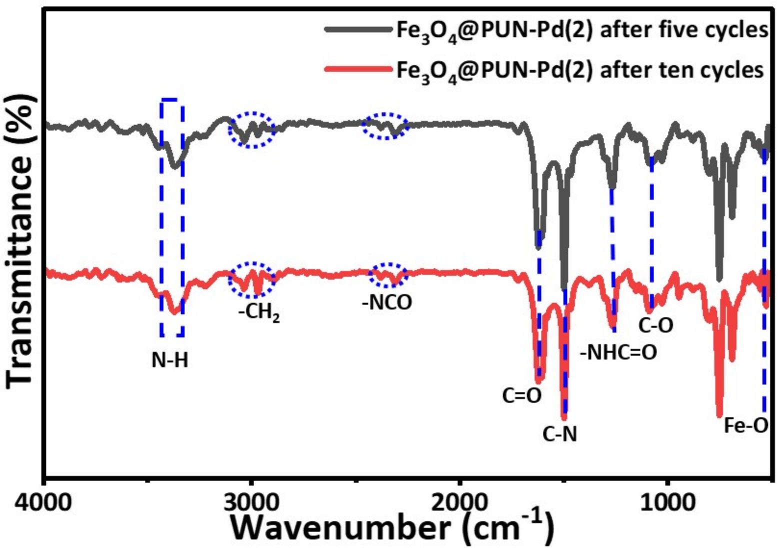

Fig. S29. FT-IR spectra of Fe_3O_4@PUN-Pd(2) after five and ten cycles.

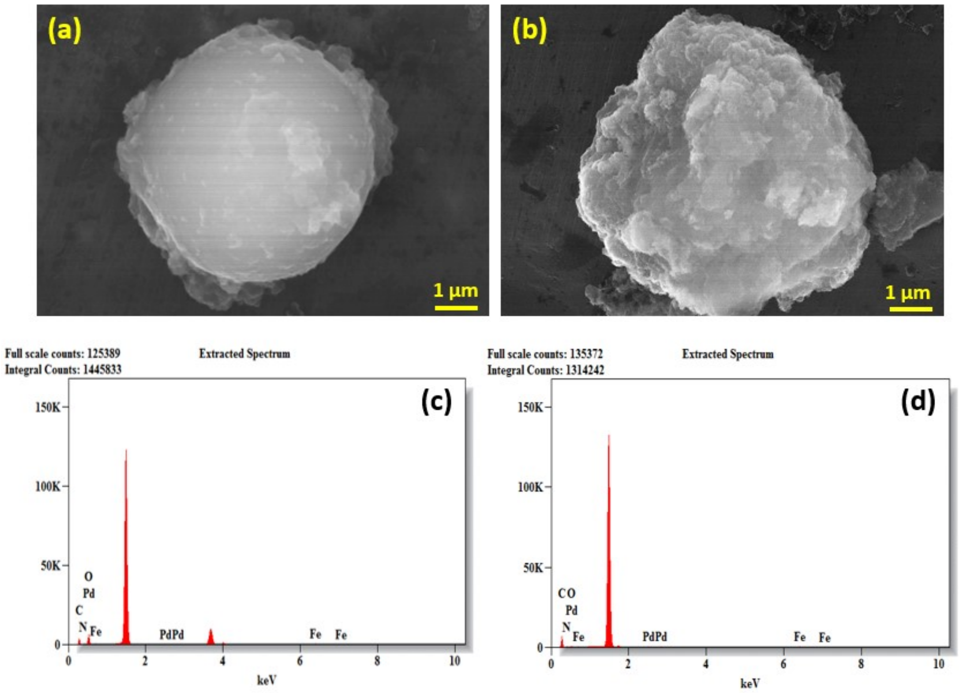

Fig. S30. SEM images and EDX spectra of Fe_3O_4@PUN-Pd(2) after 5 cycles (a,c) and 10 cycles (b,d), respectively.

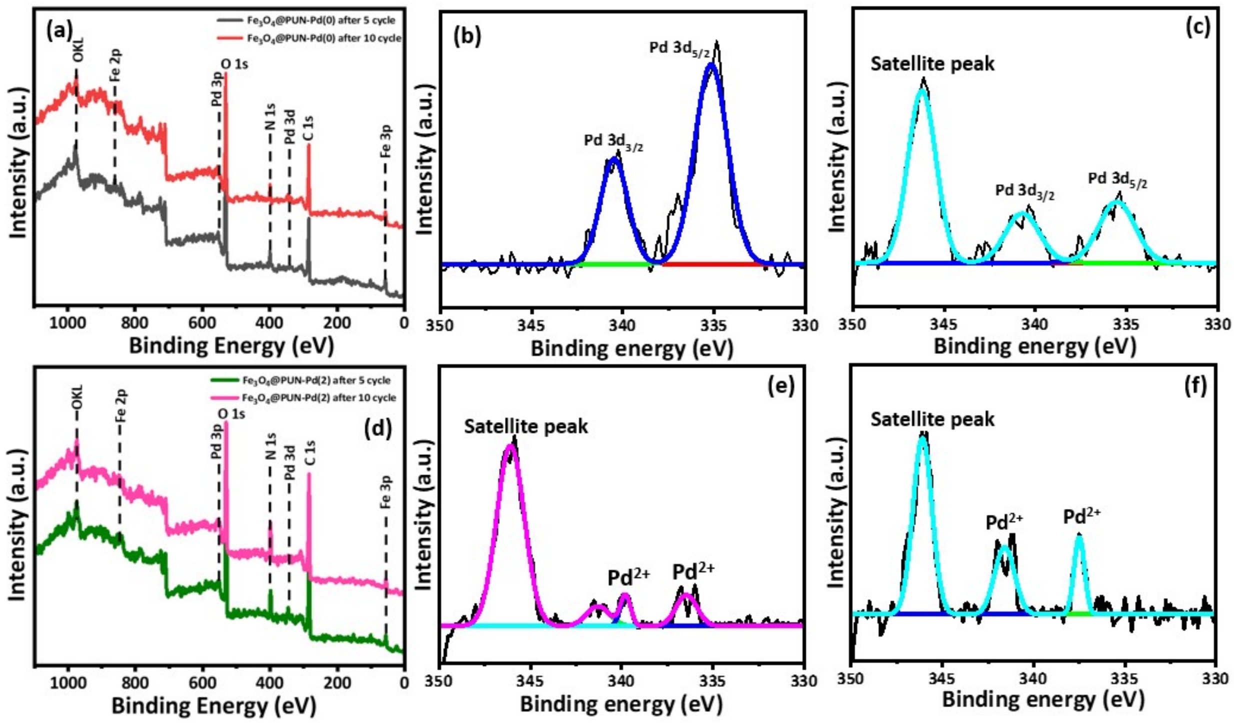

Fig. S31. (a) XPS survey spectra of Pd(0) (b) after 5 cycles and (c) after 10 cycles of Fe_3O_4@PUN-Pd(0), (d) XPS survey spectra of Pd(2) (e) after 5 cycles and (f) after 10 cycles of Fe_3O_4@PUN-Pd(2).

Table S4. Comparison of catalytic performance of Fe_3O_4@PUN-Pd(2) with other reported catalysts.

Entry	Catalyst	Metal centre	Support for the catalyst	Conversion (%)	TOF (h^{-1})	Recyclability	Reference
1	Pd@PUN	Pd	Trimethylolpropane functionalized PUN	~100	-	4	[1]
2	M/CH-PUS	Ag, Cu, Co, Ni	Chitosan (CH)-coated PUN sponge (PUS)	~100	-	3	[2]
3	Ag-PU-S/Alg	Ag	PUN (PU)-sodium alginate (S/Alg)	89.27	-	-	[3]
4	Pd–PAAm@Fe_3O_4	Pd/MNP	Polyacrylamide (PAAm)	100	-	5	[4]
5	PDOPA/PAAm@mag-rGO	Pd/MNP	Polydopamine (PDOPA)/polyacrylamide-reduced graphene oxide (rGO)	100	6738	5	[5]
6	MNP@polymer-Pd	Pd/MNP	The homopolymers and copolymers (poly(poly(ethylene glycol) methacrylate), poly(2-dimethylamino)ethyl methacrylate, and poly(2-diethylamino)ethyl methacrylate)	99	391.2	8	[6]
7	Fe_3O_4@ CMC/PDEAEMA-Pd(100)	Pd/MNP	Carboxymethyl chitosan (CMC) and poly(2-(diethyl amino)ethyl methacrylate (PDEAEMA)	> 99	386	6	[7]
8	MNP2-Pd-MC	Pd/MNP	Cellulose nanocrystal	100	305.82	7	[8]
9	Pd–Fe_3O_4@KLN	Pd/MNP	Kaolin (KLN)	100	-	5	[9]
10	Pd NPs@CHI	Pd	Cellulosic protic ionic liquids hydrogel (CHI)	99	25.14	10	[10]

11	Pd/CNT/Fe_3O_4/GO	Pd/MNP	Graphene oxide/carbon nanotube (CNT)	-	8.22× 10^{-5}	-	[11]
12	Pd/3D-AC	Pd	A hierarchically porous 3D-activated carbon (3D-AC)	100	100.8	10	[12]
13	Fe_3O_4@PUN-Pd(2)	Pd/MNP	Triazine-containing PUN	97	257	10	This work

References

1 X. Yang, X. Jiang, M. S. Bashir and X. Z. Kong, *Ind. Eng. Chem. Res.*, 2020, **59**, 2998–3007.

2 M. S. J. Khan, T. Kamal, F. Ali, A. M. Asiri and S. B. Khan, *Int. J. Biol. Macromol.*, 2019, **132**, 772–783.

3 K. Naseem, Q. Wakeel Manj, S. Akram, S. Shabbir, A. Noor, Z. H. Farooqi, S. Urooge Khan, M. Ali, M. Faizan Nazar, S. Haider and K. Alam, *Spectrochim. Acta Part A Mol. Biomol. Spectrosc.*, 2024, **317**, 124450.

4 C. Yavuz and N. Karakoyun, *Mater. Res. Bull.*, 2026, **194**, 113722.

5 N. Karakoyun, A. Zengin and M. H. Karagöz, *ChemistrySelect*, 2025, **10**, e202501104

6 U. Mahanitipong and M. Rutnakornpituk, *Polym. Int.*, 2022, **71**, 1119–1126.

7 U. Mahanitipong, S. Chanthip and M. Rutnakornpituk, *J. Inorg. Organomet. Polym. Mater.*, 2023, **33**, 1716–1728.

8 J. Xu, Y. Wang, W. Zhao, L. Han and K. C. Tam, *Mater. Today Commun.*, 2022, **33**, 104349.

9 N. Karakoyun, *Int. J. Chem. Technol.*, 2025, **9**, 306–322.

10 X. Li, F. Dong, L. Zhang, Q. Xu, X. Zhu, S. Liang, L. Hu and H. Xie, *Chem. Eng. J.*, 2019, **372**, 516–525.

11 L. K. Parrott and E. Erasmus, *RSC Adv.*, 2020, **10**, 32885–32896.

12 F. Shu, J. Wu, G. Jiang, Y. Qiao, Y. Wang, D. Wu, Y. Zhong, T. Zhang, J. Song, Y. Jin, B. Jiang and H. Xiao, *Sep. Purif. Technol.*, 2022, **300**, 121823.
